# Colossal Negative Area Compressibility in the Ferroelastic Framework Cu(tcm)

**DOI:** 10.1021/jacs.5c02999

**Published:** 2025-05-14

**Authors:** Muzi Chen, Hanna L. B. Boström, Dominik Daisenberger, Nicholas P. Funnell, Christopher J. Ridley, Mohamed Mezouar, Claudia Weidenthaler, Andrew B. Cairns

**Affiliations:** † Department of Materials, 4615 Imperial College London, Royal School of Mines, Exhibition Road, London SW7 2AZ, U.K.; ‡ London Centre for Nanotechnology, Imperial College London, London SW7 2AZ, U.K.; § Department of Chemistry, 7675 Stockholm University, Svante Arrhenius väg 16C, Stockholm SE-106 91, Sweden; ∥ Wallenberg Initiative Materials Science for Sustainability, Department of Chemistry, Stockholm University, Stockholm SE-114 18, Sweden; ⊥ 120796 Diamond Light Source Ltd., Harwell Campus, Didcot OX11 0DE, U.K.; # ISIS Neutron and Muon Source, Rutherford Appleton Laboratory, Harwell Campus, Didcot OX11 0QX, U.K.; ∇ 55553 European Synchrotron Radiation Facility (ESRF), 71, avenue des Martyrs, Grenoble Cedex 9 CS 40220, 38043, France; ○ Department of Heterogeneous Catalysis, 28314 Max-Planck-Institut für Kohlenforschung, Kaiser-Wilhelm-Platz 1, Mülheim an der Ruhr 45470, Germany

## Abstract

Copper­(I) tricyanomethanide, Cu­(tcm), is a flexible framework material that exhibits the strongest negative area compressibility (NAC) effect ever observed—a remarkable property with potential applications in pressure sensors, artificial muscles, and shock-absorbing devices. Under increasing pressure, Cu­(tcm) undergoes two sequential phase transitions (tetragonal → orthorhombic → monoclinic): It has an initial tetragonal structure (*I*4_1_ *md*) at ambient conditions, but this structure only persists within a narrow pressure range; at 0.12(3) GPa, a pressure-induced ferroelastic phase transition occurs, transforming Cu­(tcm) into a low-symmetry orthorhombic structure (*Fdd*2). The orthorhombic phase has a NAC of −108(14) TPa^–1^ in the **b–c** plane between 0.12(3) and 0.93(8) GPa. The NAC behavior is associated with framework hinge motion in a flexible framework with “wine-rack” topology. At 0.93(8) GPa, Cu­(tcm) undergoes a second phase transition and transforms into a layered monoclinic structure (*Cc*) with topologically interpenetrating honeycomb networks. The monoclinic phase of Cu­(tcm) exhibits a slight negative linear compressibility (NLC) of −1.1(1) TPa^–1^ along the *a* axis and a zero area compressibility of *K*_ac_ = *K*_a_ + *K*_c_ = 0.0(4) TPa^–1^ in the **a**–**c** plane over the pressure range of 0.93–2.63 GPa. In contrast to the orthorhombic phase, its mechanism is understood as the pressure-driven dampening of layer “rippling,” which acts to increase the cross-sectional area of the layer at higher hydrostatic pressures. These findings have implications for understanding the underlying mechanism of NAC phenomenon in framework materials.

## Graphical abstract

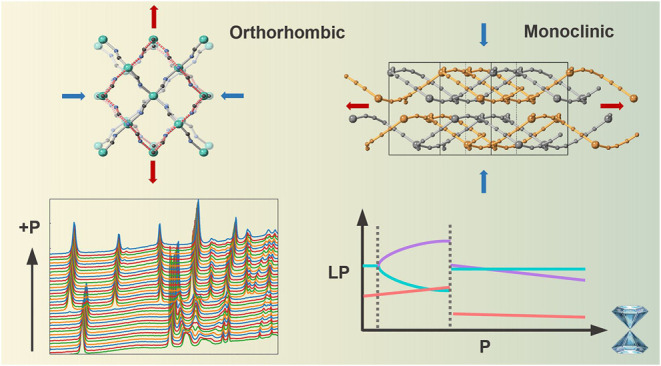

## Introduction

Recently, materials with negative compressibility (NC) properties have attracted considerable attention due to their potential applications in designing next-generation micro- or nanoscale pressure sensors, artificial muscles, and actuators.^1^ NC is a unique and extreme manifestation of pressure-induced mechanical anisotropy. When subjected to pressure, materials with this property respond counterintuitively by expanding rather than contracting in at least one linear principal direction. Based on the number of expanding directions, NC can be divided into negative linear compressibility (NLC) and negative area compressibility (NAC), which refer to uniaxial and biaxial expansion, respectively.^2^ To date, researchers have focused mainly on developing NLC materials, and they have achieved considerable success in this field. A variety of novel NLC materials has been identified, including inorganic oxides, metal cyanides, and metal–organic frameworks (MOFs)^3–5^

1 $$K_{l}=-\frac{1}{l}{\left(\frac{\partial l}{\partial P}\right)}_{T}$$

Remarkably, some of these materials demonstrate NLC effects that are several orders of magnitude stronger than the positive linear compressibility (PLC) found in conventional materials. The PLC of a general crystalline material, defined by eq 1, lies in the range 5–20 TPa^–1^.^6^ In contrast, certain coordination polymers with flexible frameworks can exhibit NLC effects with *K*_*l*_ values as high as −75 TPa^–1^.^7–9^ Thermodynamic laws dictate that the intrinsic volume of materials should decrease under hydrostatic compression.^10^ Therefore, the magnitude of NLC is significantly influenced by the volume compressibility of a material, which is inversely related to its bulk modulus (*B*_0_). Soft materials, characterized by low *B*_0_, typically possess extensive intraframework space and flexible components. These properties facilitate rapid atomic-scale framework deformation, leading to significant mechanical anisotropy under compression. However, for the same reason, they are unable to maintain their initial state over a wide range of pressures, experiencing structural collapse at relatively low pressures.

In addition to their structural flexibility, NLC materials have another distinctive feature: their structures typically exhibit specific topologies, such as “wine-rack” or “honeycomb” motifs.^11–14^ This characteristic allows for hinge motion within the framework. Examples include Zn­[Au­(CN)_2_]_2_ and Ag_3_[Co­(CN)_6_].^8,9^ Both compounds exhibit rapid contraction in certain directions under compression, facilitated by the weak metallophilic interaction between the metal cations. The significant PLC is accompanied by NLC in the perpendicular direction due to the hinged network.

Some materials have crystal structures at atmospheric pressure that exhibit NLC when pressure is applied, while others exhibit NLC only after undergoing a pressure-induced phase transition. In materials like TeO_2_ and diamondoid Zn­(CN)_2_, their initial high-symmetry cubic structures prevent framework hinge motion. However, after undergoing ferroelastic phase transitions, these materials can develop NLC properties.^15–17^ This transition, characterized by a reversible change in crystal symmetry, is induced by the application of external stress or temperature variations. Geometrically, this process can be viewed as a change from a square to a rhombic shape (Figure 1), quantified by the ferroelastic order parameter, ε

2 $$\varepsilon =\frac{\left(b-a\right)}{\left(a+b\right)}$$

where *a* = *b* represents the high-symmetry paraelastic state, and *a* ≠ *b* indicates the low-symmetry ferroelastic state. In the low-symmetry phase, the framework is released from its initial structural constraints. Upon further compression, it deforms anisotropically, expanding in some directions while contracting in others, manifesting NLC behavior.^18,19^

**Figure 1:**
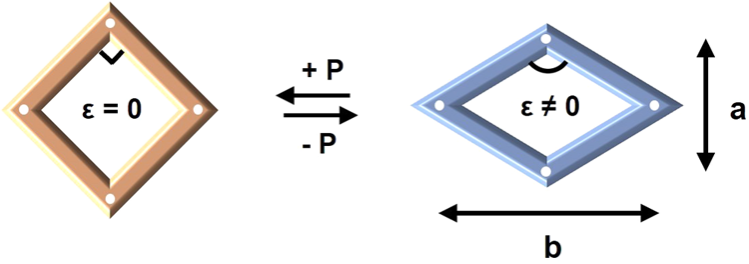
(left) Paraelastic state of the high-symmetry square frame; (right) ferroelastic state of the low-symmetry rhombic frame. The ferroelastic order parameter ε measures the extent of symmetry breaking. Reproduced with permission.^2^ Copyright 2015, Royal Society of Chemistry.

The material of focus in this research, copper­(I) tricyanomethanide (Cu­[C­(CN)_3_]), abbreviated as Cu­(tcm), has been previously shown to exhibit negative thermal expansion (NTE) properties in its low-symmetry orthorhombic phase, with an NTE coefficient of α_*b*_ = –407(28) MK^–1^ over a temperature range of 20–290 K.^20^ This value is extraordinary, exceeding four times the threshold for what is considered a “colossal” NTE (α = −100 MK^–1^).^21^ Since empirical evidence suggests that NTE and NC typically coexist, this material is expected to exhibit a significant negative structural response to pressure in the same direction.^21–23^

Our results show that Cu­(tcm) adopts a tetragonal structure (*I*4_1_ *md*) under ambient conditions. However, when external pressure is applied, it undergoes a ferroelastic phase transition to a lower symmetry orthorhombic structure (*Fdd*2). This transition results in symmetry breaking and the emergence of NAC behavior within its **b–c** plane.

The NAC effect, considered an even more extreme pressure response than NLC, has been observed in only a handful of compounds (see Table 1). Its mechanism, similar to that of NLC, involves redirecting rapid compression in a specific direction to cause expansion in the perpendicular directions. Under hydrostatic pressure, NAC materials increase their surface area, enabling them to act as substrates that can provide orders of magnitude amplification of piezoelectric response. Many known NAC materials are characterized by a 2D layered structure with weak bonding along the stacking direction.^24–26^ Upon exposure to pressure, rapid compression along this direction leads to structural reconfigurations within the layers, coupled with lattice expansion along the direction of the layer plane. Despite the absence of a typical layered structure, Cu­(tcm) exhibits an intriguing open interpenetrating structure (Figure 2). This configuration provides considerable space within the framework, facilitating rapid compression of the unit cell in a particular direction. Such a distinctive feature establishes Cu­(tcm) as a prime candidate for NAC with potential applications in pressure sensors, data storage devices, microelectromechanical systems (MEMS), and actuators.^27–30^

**Table 1:** Review of Compressibilities for NAC Materials Based on Existing Literature

material	range (GPa)	*K*_1_ (TPa^–1^)	*K*_2_ (TPa^–1^)	*K*_3_ (TPa^–1^)	*K*_NAC_ (TPa^–1^)	*B*_0_ (GPa)	structural type
Zn(CH_3_COO)_2_·2H_2_O^24^	0.15–4.44	40.4(15)	–2.1(4)	–6.0(7)	–8.1(8)	20(2)	2D layers
CrAs^31^	0.18–0.35	–7.98	38.4	–3.71	–11.69		distorted octahedral network
[Zn(L)_2_(OH)_2_]_n_·H_2_O^32^	1.0–2.4	145(9)	–36(3)	–36(3)	–72(6)	3.5(8)	helices
[Zn(L)_2_(OH)_2_]_n_·MeOH^32^	1.3–4.01	25.7(13)	–2.3(11)	–2.3(11)	–4.6(2)	22.8(25)	helices
Ag(tcm)^25^	0–0.615	–3.5(6)	66(20)	–4.0(6)	–7.5(8)		2D layers
Ag_2_C_2_O_4_ ^33^	0–0.848	61.36	–16.73	–19.98	–36.71		edged shared chain
KBe_2_BO_3_F_2_ ^26^	0.22–6.39	–0.5(1)	–0.5(1)	22.1(13)	–1.0(2)		2D layers
NaV_2_O_5_ ^3^	4–10	–0.2	–1.3	15.6	–1.5	24(3)	2D layers
2–MeBzlm^34^	0.26–1.36	90(2)	–9(7)	–27(3)	–36(10)	7.6(5)	chain structure

**Figure 2:**
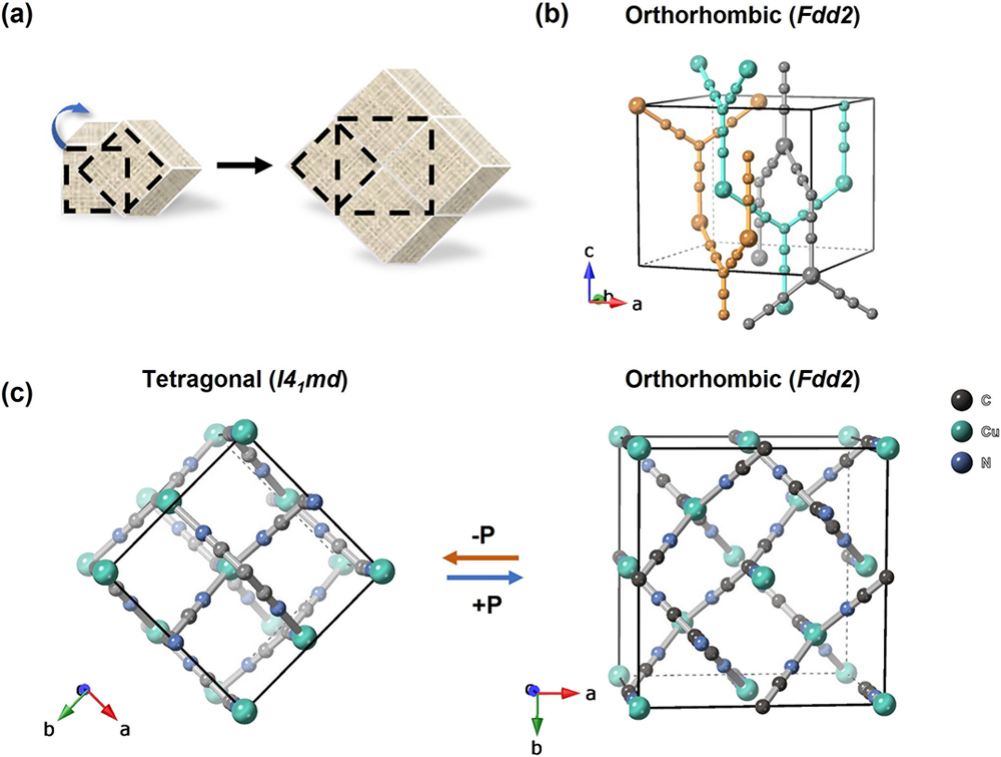
(a) Schematic illustration showing the transformation from a tetragonal unit cell (left) to a larger orthorhombic unit cell (right, indicated by dashed lines). (b) Crystal structure showing the three-dimensional interpenetrating network in the orthorhombic (*Fdd*2) phase, with different colors highlighting the interpenetrating networks. (c) Pressure-induced phase transition from tetragonal *I*4_1_ *md* structure (left) to orthorhombic *Fdd*2 structure (right). This transformation involves both a change in unit cell symmetry and a cooperative tilting of the *tcm* ^–^ ligands.

To investigate this hypothesis, Cu­(tcm) was crystallographically characterized under varying pressure conditions through multiple high-pressure diffraction measurements. The results show that Cu­(tcm) undergoes two symmetry-lowering phase transitions within a pressure range of 0–3.82 GPa, transforming its structure from tetragonal to orthorhombic, and then to monoclinic. In addition, the linear compressibility (*K*_*l*_) in each direction was calculated from the relationship between lattice parameters and pressures. Given the rapid and multiple structural changes Cu­(tcm) experiences upon compression, the chosen pressure step and range strongly influence the *K*_*l*_ values obtained. As a result, the *K*_*l*_ values calculated from four separate experiments (X1, X2, X3: synchrotron X-ray diffraction; N1: neutron diffraction) are comparable but not entirely consistent. Analysis of the two data sets with the widest pressure range and finest pressure steps (N1 and X3) reveals that the orthorhombic phase of Cu­(tcm) has an NLC of −93(13) TPa^–1^ in the ***b*** direction and −14.9(11) TPa^–1^ in the ***c*** direction, giving a total NAC of −108(14) TPa^–1^ in the **b**–**c** plane. With a NAC value that is an order of magnitude larger than [Zn­(L)_2_(OH)_2_]·*n*H_2_O (L = 4-(1*H*-naphtho­[2,3-*d*]­imidazol-1-yl)­benzoate) (*K*_ab_ = −72(6) TPa^–1^), and 2 orders of magnitude larger than that of Ag­(tcm) (*K*_ac_ = −7.5(8) TPa^–1^), Cu­(tcm) shows an exceptional NAC performance not previously observed in other materials.^25,32^

The significant NAC magnitude of Cu­(tcm) compared to other materials can be attributed to its distinct structural features. Despite sharing the same tcm^–^ ligand, Ag­(tcm) and Cu­(tcm) adopt different framework topologies due to the different ionic radius of the central metal ions. While Ag­(tcm) has a layered structure where NAC arises from the dampening of layer “rippling”, Cu­(tcm) adopts a “wine-rack” like framework that allows for more dramatic hinge motion and thus enhanced NAC. In addition, unlike [Zn­(L)_2_(OH)_2_]·*n*H_2_O, Cu­(tcm) has a framework without guest molecules that might otherwise provide steric resistance to framework deformation. Thus, compared to the former, Cu­(tcm) has a larger NAC and also greater consistency and reliability between different synthesis batches.

## Results and Discussion

Cu­(tcm) adopts a tetragonal structure (*I*4_1_ *md*) at low pressures (0–0.12(3)­GPa), with Cu atoms tetrahedrally coordinated by tcm ligands via terminal N atoms. The stability of this phase within such a narrow pressure range makes detailed investigation of its pressure response challenging. Additionally, the tetragonal phase of Cu­(tcm) behaves as a highly symmetric paraelastic state with a relatively rigid “wine-rack” like framework, making it less sensitive to pressure. These factors, combined with measurement uncertainties in both lattice parameters from diffraction patterns and pressure calibrations, influence the determination of its compressibility. We have made several attempts (X2, X3 and N1) to determine its compressibility, but the results vary widely ( Table S1). The challenge lies primarily in precisely controlling the pressure within this narrow regime, which is particularly demanding in both diamond anvil cells (DACs) and the Paris-Edinburgh press.

At 0.12(3)­GPa, Cu­(tcm) ferroelastically transforms into an orthorhombic structure (*Fdd*2) of lower symmetry, which persists up to 0.93(8)­GPa. This change represents a displacive transition associated with the softening of a zone-center mode, preserving the overall connectivity and periodicity of the structure. Specifically, the transition involves tilting of tcm^–^ units about the *c* axis, with each tcm^–^ unit maintaining its triangular arrangement of three N1–C≡N2 node without distortion of the C–N1–C angle. The interpenetrating structure of Cu­(tcm) provides substantial intraframework space to accommodate these tilts, which induce displacement of the connecting Cu^+^ cations and consequent reorientation of the framework (see Figure 2). Upon pressure release, the material returns to its initial tetragonal phase.

The orthorhombic structural model of Cu­(tcm) was first introduced by Hunt et al. in their study of its low-temperature behavior.^20^ In that work, the authors observed significant anisotropic peak broadening in their diffraction patterns at room temperature, which intensified upon cooling and later split into two separate peaks. This peak broadening was attributed to symmetry lowering beyond *I*4_1_ *md*. Our research is consistent with these findings, as we have also observed similar anisotropic peak broadening in our diffraction patterns (see Figure 3). When studying the structural transformation, the tetragonal *a* lattice parameters are normalized according to eq 3 to facilitate direct comparison with the orthorhombic phase

3 $$a^{\prime }=a\times \sqrt{2}$$

**Figure 3:**
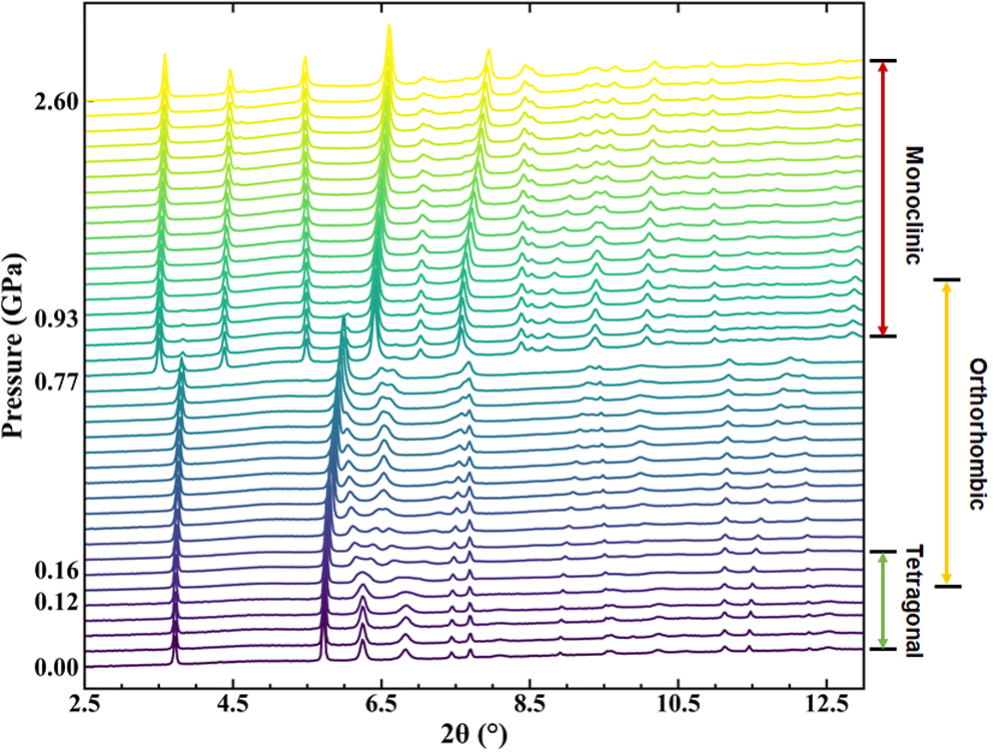
HP-PXRD patterns of Cu­(tcm) measured at Diamond Light Source (X3) using radiation with a wavelength of 0.3757 Å. The patterns represent, in ascending order, the tetragonal (0–0.12 GPa), orthorhombic (0.12–0.93 GPa), and monoclinic phases (0.77–2.60 GPa).

Using these normalized parameters, we find that the transformation to the orthorhombic *Fdd*2 structure exhibits a continuous trend in *V*/*Z* (unit-cell volume per formula unit) across the transition pressure, which is characteristic of a second-order phase transition. This continuous nature is quantitatively confirmed by our strain-order parameter analysis shown in Figure 4(c), where the spontaneous strain *ε* (as defined in eq 2) emerges continuously from zero at the critical pressure and follows the relationship:

4 $$\varepsilon =\kappa |P-P_{T}{|}^{0.5}$$

**Figure 4:**
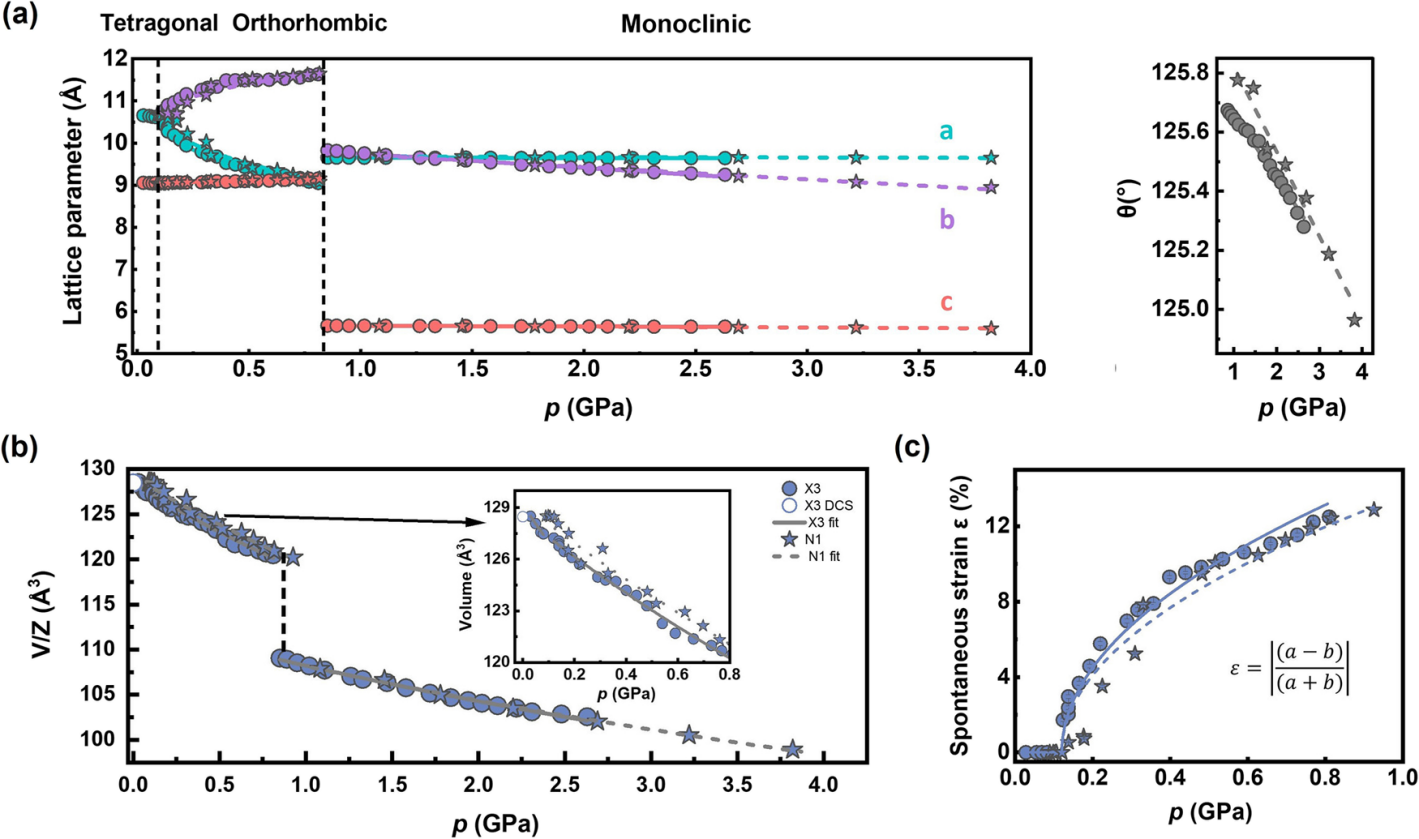
(a) The pressure-dependent behavior of the lattice parameters across the three phases of Cu­(tcm) is analyzed using the X3 and N1 data sets. The X3 data are shown as solid circles, while the N1 data are shown as stars. The lattice parameters *a*, *b* and *c* are represented by different colors, and the angle β is shown for the monoclinic phase. The fitted lines are dashed for the stars and solid for the circles. The data points for the tetragonal and monoclinic phases were subjected to a weighted linear fit, while the orthorhombic data were fitted using an exponential eq 5. (b) The volume of Cu­(tcm) against pressure at ambient temperature, fitted using the second-order BM EOS (eq 6). (c) The pressure variation of the spontaneous strain, as defined by eq 2. They have been fitted using eq 4. These fits are shown as solid and dashed lines, respectively.

Here, ε represents the strain induced by the applied pressure *P*, κ is a proportionality constant, and *P*_T_ denotes the transition pressure.^20^ This square-root power law dependence is precisely what Landau theory predicts for a second-order transition, where the order parameter grows continuously from the transition point. The data in Figure 4(c) also demonstrates proper ferroelasticity, where strain itself acts as the primary order parameter, rather than improper ferroelasticity where other parameters such as magnetization or electric polarization would drive the transformation.^35–37^

In contrast to the tetragonal phase, the orthorhombic phase of Cu­(tcm) exhibits a pronounced sensitivity to pressure. Upon compression, the unit cell undergoes a significant anisotropic deformation manifesting as NAC. This deformation is characterized by a substantial contraction of the *a*–axis lattice parameter, while the *b*– and *c*–axes expand. The cause of this anomalous compression behavior in the orthorhombic phase of Cu­(tcm) has been linked to hinge motion within a “wine-rack” framework. This mechanism emphasizes the characteristic changes in the shape of the framework under external stress, which manifests as elongation in one specific direction and contraction in another. (see Figure 5(a)) Once the external pressure is removed, the structure reverts to its original shape.^38^

**Figure 5:**
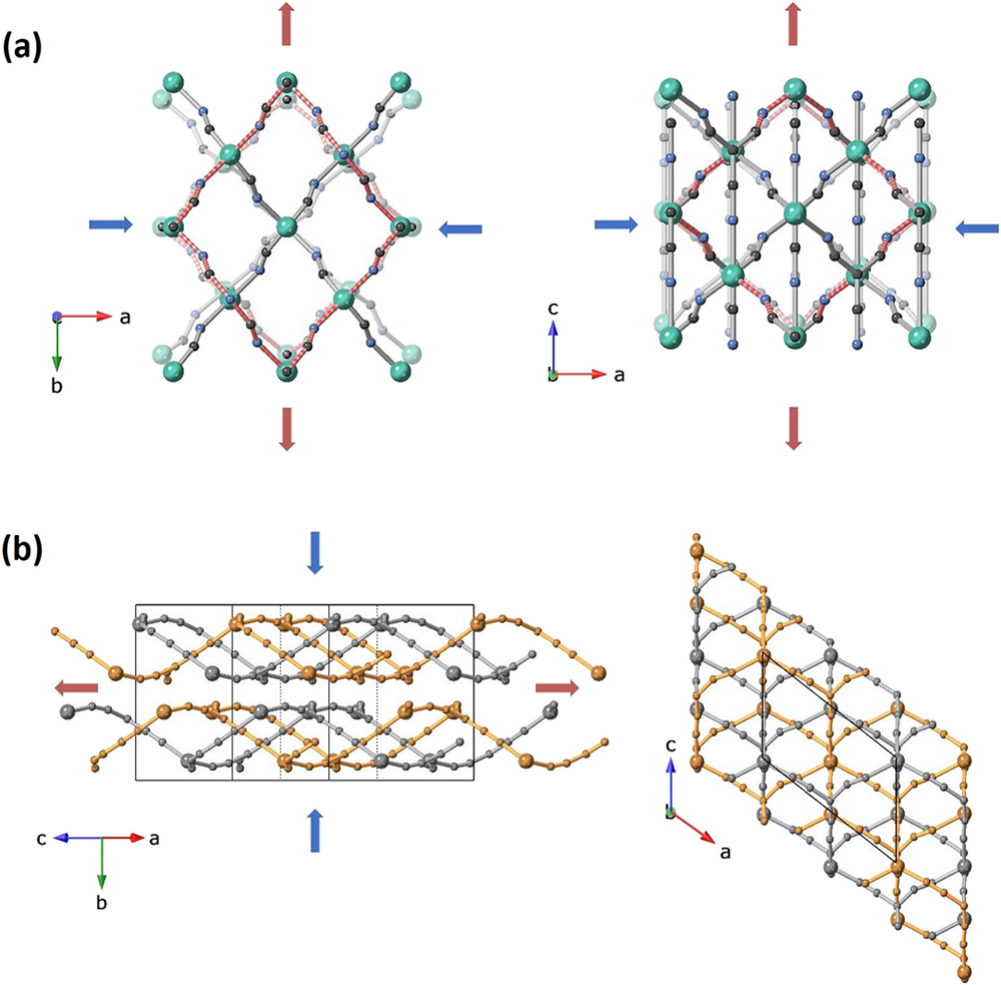
(a) Schematic representation of the NAC mechanism in the orthorhombic phase of Cu­(tcm). Orthorhombic Cu­(tcm) has a topology with a high degree of freedom resembling a wine rack. When it is compressed, rapid contraction along the *a* axis is transformed into expansion in the perpendicular directions via flexing of the framework. (b) Representation of the layered structure with topological double interpenetrating honeycomb networks of the monoclinic phase of Cu­(tcm). The nearly zero area compressibility mechanism in its **b–c** plane is understood as the pressure-driven dampening of layer “rippling,” which acts to increase the layer cross-sectional area at higher hydrostatic pressures.

The framework hinge motion observed in Cu­(tcm) stems from its distinctive structural compositions, which feature both rigid and nonrigid components. Both Cu­(I) cations and tcm^–^ ligands act as rigid nodes, conferring stability to the overall framework. In contrast, their junctions act as nonrigid parts, imparting dynamic flexibility to the structure. This combination of stiffness and flexibility allows the network to exhibit hinge motion under pressure. In addition, Cu­(tcm) has a three-interpenetrating structure organized by Cu^+^ and tcm^–^ ions which are linked to each other in the form of trigonal nodes. The distinctive configuration of this structure offers ample intraframework space, leading to pronounced compressibility along *a*–axis directions. The rapid compression translates into substantial expansion along the perpendicular *b*– and *c*–axes via flexing of the framework.

The linear compressibilities of Cu­(tcm) along three principal axes at different pressures were calculated using the Pascal software, and the calculation results are shown in SI Table S1.^39^ In general, the midpoint value is used to describe the linear compressibility of a material. This suggests that the selected pressure range and the number of data points have a significant impact on the linear compressibility value of soft materials. In the case of Cu­(tcm), the calculations of X1 and X2 data yield respective NAC values of −45(7) TPa^–1^ over the pressure range of 0.19–0.84 GPa and −146(46) TPa^–1^ over the pressure range of 0.09–0.56 GPa. They are vastly different due to the fact that the linear compressibility of Cu­(tcm) varies significantly with pressure, demonstrating the importance of choosing the appropriate pressure range when determining linear compressibility. Thus, small pressure steps and a wide pressure range were used to generate a more precise NAC value in N1 and X3. According to the findings, Cu­(tcm) exhibits remarkable NLC in the direction of the *b* and *c* lattice parameters with values of −93(13) and −14.9(11) TPa^–1^, respectively, yielding an overall NAC value of −108(14) TPa^–1^ over a pressure range of 0.12(3)–0.93(8) GPa. The colossal NAC value in the **b–c** plane causes an even larger PLC along the perpendicular *a*–axis, which possesses a magnitude of 156(14) TPa^–1^. Due to the fact that Cu­(tcm) has a tremendous PLC that exceeds the volume compressibility, it could be suitable for application in molecule-scale strain amplifiers.^27^

The NAC behavior in Cu­(tcm) occurs over the pressure range of 0.12–0.93 GPa. This range is comparable to other molecular NAC materials—Ag­(tcm) exhibits NAC only within 0–0.615 GPa while [Zn­(L)_2_(OH)_2_]*_n_* ·H_2_O shows NAC between 1.0–2.4 GPa.^25,32^ It represents a specific operational window that offers valuable opportunities for potential applications. This sub-GPa pressure range is particularly relevant for precision sensing technologies and mechanical systems operating in moderate pressure environments. With its exceptional NAC magnitude (−108(14) TPa^–1^), Cu­(tcm) shows great promise for applications in efficient pressure sensors, mechanical amplifiers, and actuators for precision electronic devices such as touchscreens and tactile interfaces.

However, it is important to note that our current study is limited to powder samples of Cu­(tcm), as growing single crystals has proven challenging despite extensive efforts. This limitation presents obstacles for direct technological implementation of the applications described above, which would benefit from larger single crystals or thin films with controlled orientation for optimal device performance. The powder form still allows fundamental characterization of the material’s exceptional mechanical properties, but future work focused on developing reliable methods for growing Cu­(tcm) single crystals will be critical. Such advances would enable practical devices that could fully utilize the remarkable NAC behavior in real-world sensing and actuation applications.

With increasing pressure, Cu­(tcm) undergoes a second symmetry change at 0.93(8)­GPa, transforming from an orthorhombic to a monoclinic structure. The discontinuity in the *V*/*Z* vs *P* plot (Figure 4(b)) identifies this as a first-order phase transition. Volume data for both orthorhombic and monoclinic phases were fitted using eq 6 through EoSFit7-GUI software, yielding bulk moduli of 10.6(3) GPa and 21.2(6) GPa, respectively, which indicates typical strain hardening under pressure.^40^ Since the pressure–volume data for these phases do not begin at ambient pressure, these values represent the bulk moduli at the onset pressures of each phase (*B*_0.12_ and *B*_0.93_) rather than zero-pressure bulk moduli (*B*_0_). Rietveld refinement of an ISODISTORT-predicted *Cc* model was employed to solve the high-pressure structure of Cu­(tcm).^41^ The result reveals that the monoclinic phase of Cu­(tcm) has a layered structure with double interpenetrating honeycomb networks, similar to the structure of Ag­(tcm) at ambient conditions.^25^ Both structures feature stacked (6,3)-nets where metal ions and tcm^–^ ligands serve as trigonal nodes, though with different metal coordination environments (see Figure S14 in SI for detailed structural comparison).

In comparison, Ag­(tcm) displays a NAC of *K*_(ac)_ = *K*_a_ + *K*_c_ = −7.5(8)­TPa^–1^ over the pressure range of 0–0.615 GPa.^25^ Its mechanism is understood as the pressure-driven dampening of layer “rippling,” which acts to increase the layer cross-sectional area at higher hydrostatic pressures.^25^ Our structural analysis of monoclinic Cu­(tcm) confirms similar behavior, as evidenced by the elastic modulus data. The monoclinic phase shows PLC along the *b* and *c* axes with values of 31.5(5) and 1.1(3)­TPa^–1^ respectively, while exhibiting slight NLC along the *a* axis at −1.1(1)­TPa^–1^ over the pressure range of 0.93–2.63 GPa. The combination of slight NLC along a and minimal PLC along *c* results in a zero area compressibility of *K*_(ac)_ = *K*_a_ + *K*_c_ = 0.0(4)­TPa^–1^ in the **a**–**c** plane. Although both compounds show pressure-driven layer rippling dampening, Cu­(tcm) maintains a constant cross-sectional area in the **a**–**c** plane during compression, in contrast to the expanding area observed in Ag­(tcm). This difference in mechanical behavior may be related to their distinct crystal symmetries, as Ag­(tcm) adopts a higher symmetry orthorhombic space group *Ima*2 compared to the monoclinic *Cc* symmetry of Cu­(tcm).

Notably, all pressure-induced phase transitions in Cu­(tcm) are fully reversible. Whether compressed to the orthorhombic phase (0.12–0.93 GPa) or further to the monoclinic phase (>0.93 GPa), Cu­(tcm) returns to its original tetragonal structure upon complete decompression to ambient conditions. This reversible behavior allows the material to reset after pressure cycling, which could be advantageous for applications requiring repeated pressure sensing capabilities.

## Conclusions

Cu­(tcm) has an initial tetragonal structure with a “wine-rack” topology at ambient conditions. It undergoes a ferroelastic phase transition and transforms into an orthorhombic structure at 0.12(3) GPa. This transformation is accompanied by a tilting of the trigonal tcm^–^ units, resulting in a significant increase in orthorhombic lattice parameters (while the normalized volume maintains its expected decrease under compression). In contrast to the tetragonal phase, the orthorhombic phase of Cu­(tcm) possesses a ferroelastic hinged “wine-rack” framework with high flexibility. It undergoes dramatic anisotropic deformation upon compression: Cu­(tcm) has a NAC value of −108(14) TPa^–1^ in the **b–c** plane and a PLC value of 156(14) TPa^–1^ along the *a*–axis over the pressure range of 0.12(3)–0.93(8) GPa. This NAC value significantly exceeds the previous record holder, [Zn­(L)_2_(OH)_2_]*_n_* ·H_2_O, which exhibits a NAC of −72(6) TPa^–1^.^32^ The mechanism responsible for this deformation has been identified as a hinge motion in a “wine-rack” framework. This combination of rigid and flexible components allows the framework to deform anisotropically under pressure. At 0.93(8) GPa, Cu­(tcm) undergoes an additional first-order phase transition, changing its structure from orthorhombic to monoclinic. The monoclinic phase of Cu­(tcm) has a layered structure with topological double interpenetrating honeycomb networks. Upon compression, rapid contraction occurs along the unit cell direction of layer stacking. It is converted into an increase in the layer cross-sectional area due to the dampening of layer “rippling.” Monoclinic Cu­(tcm) therefore has zero area compressibility in the **a**–**c** plane over the pressure range of 0.93–2.63 GPa.

## Experimental Section

### Synthesis

Cu­(tcm) was prepared following the literature procedure.^20^ A 5 mL aqueous solution of NaHSO_3_ (104.0 mg, 1 mmol) was added dropwise to a vigorously stirring 2 mL aqueous solution of CuSO_4_ (159.6 mg, 1 mmol) over 30 min to achieve a green Cu­(I) solution. Then, a 10 mL aqueous solution of K­(tcm) (129.2 mg, 1 mmol) was added to the Cu­(I) solution, resulting in the immediate precipitation of an off-white solid. To ensure a complete reaction, the mixture was stirred for an additional 2 h. The obtained material was collected by centrifugation, washed with deionized water, and dried in an oven (60 °C, 1 day). X-ray diffraction (XRD) analysis confirmed the formation of Cu­(tcm).

### Structure Determination and Refinement

To systematically investigate the pressure-dependent structural behavior of Cu­(tcm), we conducted multiple high-pressure diffraction experiments using both X-ray and neutron radiation sources. These complementary techniques were employed to ensure comprehensive structural characterization and enhance data reliability. The experiments included three high-pressure powder X-ray diffraction (HP-PXRD) measurements and one high-pressure powder neutron diffraction (HP-PND) measurement, abbreviated as X1, X2, X3, and N1, respectively.

HP-PXRD experiments were conducted at two synchrotron facilities: X3 at the I15 beamline of Diamond Light Source (λ = 0.3757 Å) and X1, X2 at the ID27 beamline of ESRF (λ = 0.3738 Å). In both cases, the sample was loaded into a diamond anvil cell (DAC), with Daphne 7373 as the pressure transmitting medium (PTM) and ruby crystals for pressure calibration. X3 data were collected between 0.03 and 2.63 GPa. The ranges for X1 and X2 data collection were 0.08–2.49 and 0.08–0.56 GPa, respectively. After each experiment was completed, the sample was decompressed to observe reversibility. All obtained data were processed by Dioptas and Dawn.^42–44^

N1 was collected using the PEARL instrument at the ISIS Neutron and Muon Source.^45^ The powder sample was encapsulated in a null-scattering Ti–Zr gasket, accompanied by a Pb pressure marker and a pentane/isopentane mixture serving as the PTM. This entire assembly was loaded between a pair of anvils. Hydrostatic pressure was applied using a Paris-Edinburgh (P-E) press.^45^ The Cu­(tcm) sample was subjected to two separate experiments with different pressure ranges. The first experiment involved the application of pressures varying between 0.09 and 0.12 GPa at room temperature and the second experiment consisted of measurements in the range of 0.18 to 3.82 GPa. This two-stage experimental design allowed a thorough collection of neutron diffraction data across both low and high-pressure regions.

Rietveld refinement of high-pressure diffraction patterns was performed using TOPAS Academic v4 or v5.^46,47^ The crystal structures of Cu­(tcm) at ambient and low temperatures have been previously reported in the literature, being applicable to both the ambient tetragonal and low-pressure orthorhombic phases of Cu­(tcm).^20^ However, the structure of the high-pressure phase of Cu­(tcm) remained unresolved. To address this, an indexing method was employed in combination with ISODISTORT.^41,48^ The resulting crystal structure was then validated by Rietveld refinement.

### Linear Compressibility and Bulk Modulus

The lattice parameters were obtained through Rietveld refinement of the diffraction patterns and analyzed in conjunction with their corresponding pressures. The linear compressibility coefficients for each phase were calculated using PASCal software.^39^ The pressure-dependent evolution of lattice parameters was fitted using an improved exponential model described by eq 5

5 $$l=l_{0}+\lambda {\left(p-p_{c}\right)}^{\nu }$$

where *l* represents the lattice parameter, *l*_0_ is the initial measurement, *p* is the applied pressure, and λ and *p*_c_ are fitting-derived constants.^2^ For volume analysis, a second-order Birch–Murnaghan equation of state (BM EoS) was employed using the EoSFit7 GUI software^40^

6 $$P=\frac{3B_{0}}{2}\left[{\left(\frac{V_{0}}{V}\right)}^{7/3}-{\left(\frac{V_{0}}{V}\right)}^{5/3}\right]$$

where *P* is the pressure, *V* is the volume at pressure *P*, *V*_0_ is the initial volume at ambient pressure, and *B*_0_ is the bulk modulus.

## Supplementary Material



## Notes

Additional experimental details; crystallographic data tables; X-ray and neutron diffraction patterns with Rietveld refinements; pressure-dependent lattice parameter analysis; and compressibility calculations across multiple experimental data sets (X1-X3, N1) for all structural phases (PDF)

## ◆

Neutron Scattering Division, Oak Ridge National Laboratory, Oak Ridge, Tennessee 37831, United States
