# Supplementary material for: Colossal Negative Area Compressibility in the Ferroelastic Framework Cu(tcm)
Source: J Am Chem Soc. 2025 May 14;147(21):17946–53. doi: 10.1021/jacs.5c02999 (PMC12123623; doi:10.1021/jacs.5c02999)
Supplement: Supplementary file 1 [file ja5c02999_si_001.pdf]

SUPPORTING INFORMATION

Colossal negative area compressibility in the ferroelastic framework Cu(tcm)

Muzi Chen,[ab*] Hanna L. B. Boström,[cd] Dominik Daisenberger,[e] Christopher J. Ridley,[f] Nicholas P. Funnell,[f] Mohamed Mezouar,[g] Claudia Weidenthaler,[h] and Andrew B. Cairns[ab*]

[a] Department of Materials, Imperial College London, Royal School of Mines, Exhibition Road, SW7 2AZ, London, U.K

[b] London Centre for Nanotechnology, Imperial College London, London SW7 2AZ, UK

[c] Department of Chemistry, Stockholm University, Svante Arrhenius väg 16C, SE-106 91, Stockholm, Sweden

[d] Wallenberg Initiative Materials Science for Sustainability, Department of Chemistry, Stockholm University, SE-114 18, Stockholm, Sweden

[e] Diamond Light Source Ltd., Harwell Campus, Didcot OX11 0DE, U.K

[f] ISIS Neutron and Muon Source, Rutherford Appleton Laboratory, Harwell Campus, Didcot OX11 0QX, U.K

[g] European Synchrotron Radiation Facility (ESRF), 71, avenue des Martyrs, CS 40220, 38043 Grenoble Cedex 9, France

[h] Department of Heterogeneous Catalysis, Max-Planck-Institut für Kohlenforschung, Kaiser-Wilhelm-Platz 1, 45470 Mülheim an der Ruhr, Germany

*To whom correspondence should be addressed;

E-mail: muzichen@kofo.mpg.de

E-mail: a.cairns@imperial.ac.uk

Contents

1. Experimental Strategy 3
2. Linear Compressibility and Bulk Modulus 4
3. Crystallographic Details 6
4. X3 Data of Cu(tcm) 8
5. N1 Data of Cu(tcm) 11
6. X2 Data of Cu(tcm) 14
7. X1 Data of Cu(tcm) 17
8. Structural comparison between Ag(tcm) and Cu(tcm) 20
9. Reference 21

1. Experimental Strategy

As stated in the main text, we performed a total of four high-pressure structural measurements on Cu(tcm), which included three high-pressure powder X-ray diffraction experiments (X1, X2, and X3) and one high-pressure powder neutron diffraction experiment (N1). X1 and X2 were the first two experiments conducted among them. In X1 and X2, two pressure ranges, 0.08–2.49 GPa and 0.01–0.56 GPa, were measured using diamond-anvil cells in the European Synchrotron Radiation Facility (ESRF), respectively. The X1 experiment investigated Cu(tcm) deformation over a wide pressure range, revealing two structural phase transitions between 0.08 and 2.46 Gpa. The X2 experiment, on the other hand, investigated the notable elastic behaviour of Cu(tcm) at low pressures more thoroughly: A small pressure increment in X2 was achieved by replacing the stainless-steel gasket with a softer copper gasket and using larger diamonds for measurement. The X2 result suggests that Cu(tcm) exhibits NLC of –131(40) TPa^{-1} in the *b* direction and –11.3(5) TPa^{-1} in the *c* direction, giving an overall negative area compressibility (NAC) of –142(41) TPa^{-1} in the **b-c** plane. We are thrilled to discover that Cu(tcm) has an extreme negative compressibility that has never been observed in other materials.

However, two problems are encountered in solving the Cu(tcm) structure of ongoing interest. The first is an extra peak found in diffraction patterns that cannot be fitted in the refinement with any satisfactory model (e.g., a tetragonal structure of $I4_1md$ symmetry, with lattice parameters a = b = 7.551 (1) Å and c = 9.042 (1) Å). The possible reason for this extra peak is that the measured sample was not fresh, and a long time of storage can cause a slow phase transition in compounds. Another potential problem is that the synchrotron X-ray used in X1 and X2 was relatively high in energy, which caused damage to the sample. This is known for samples in low oxidation states and was of concern here because shadowing on the sample was observed on recovery. Beam damage to the sample may cause slow degradation of the sample, impacting the crystal structure information we get. More critically, beam damage is also known to cause lattice parameter expansion, and so to confirm the behaviour observed, it must be measured without these effects. Therefore, a variable-pressure neutron diffraction experiment (N1) on a freshly prepared Cu(tcm) sample was carried out at PEARL, ISIS. Neutron diffraction is similar to X-ray diffraction, but the different type of radiation gives complementary information. Neutrons are very penetrating and do not strongly interact with the sample, so no beam damage was expected using this technique. In addition to neutron diffraction, Cu(tcm) of the same batch was measured at the Diamond Institution using synchrotron radiation X-ray powder diffraction (X3). During this measurement, no beam damage was found. The obtained high-pressure diffraction patterns help in the determination of the high-pressure crystal structure of Cu(tcm).

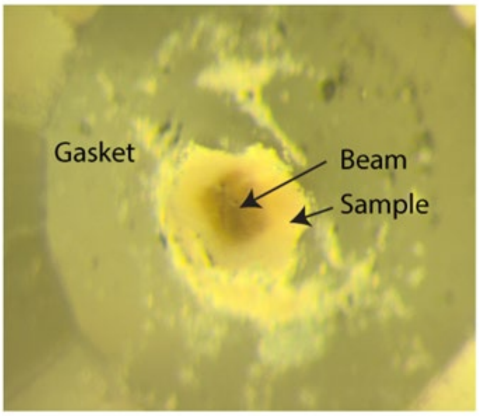

Figure S1: The Cu(tcm) sample damaged by Synchrotron X-ray with high energy.

2. Linear Compressibility and Bulk Modulus

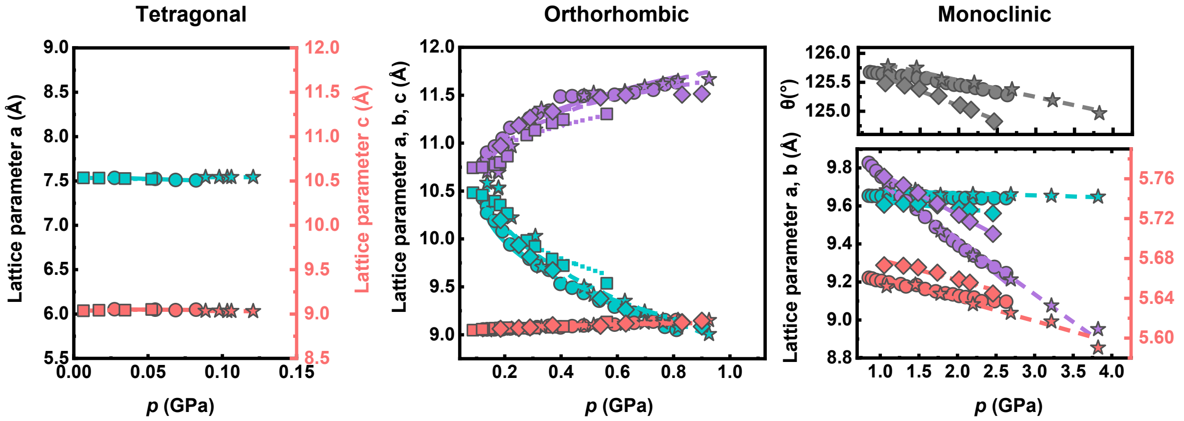

Figure S2: The pressure dependence of Cu(tcm) lattice parameters across three phases, as revealed by synchrotron radiation X-ray powder diffraction (X3, X2, X1) and time-of-flight (TOF) neutron powder diffraction (N1). Specific symbols - circles, squares, diamonds, and stars - differentiate the X3, X2, X1, and N1 data respectively. In addition, the choice of colours represents varying crystallographic directions: the *a* lattice parameter in green, *b* and *c* lattice parameters in purple and pink correspondingly. The monoclinic angle β (shown in grey) is plotted for the monoclinic phase where it deviates from 90°. The respective fitting lines follow these symbols: solid for circles, dotted for squares, dash-dotted for diamonds, and dashed for pentagrams. For the tetragonal and monoclinic phases, a weighted linear fit was applied to the data points, while the orthorhombic phase was modelled with the equation $l = l_0 + \lambda(p - p_c)^{\nu}$ (Eq S1), where l symbolises a specific directional lattice parameter, l_0 is the initial lattice value in the same direction, p denotes the measured pressure, and ν has a constant value of 0.5.[S1]

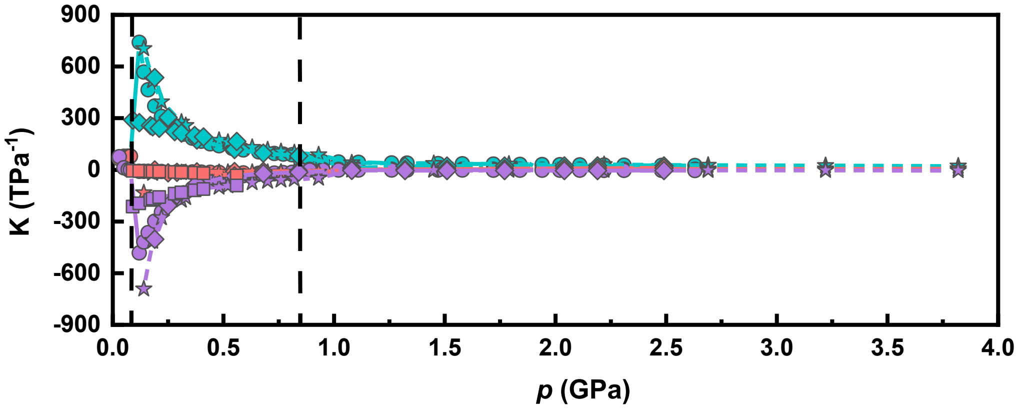

Figure S3: The pressure dependence of linear compressibility in Cu(tcm) determined with PASCal.[S2] Median values representing the linear compressibility coefficients for each phase are displayed in Table S1.

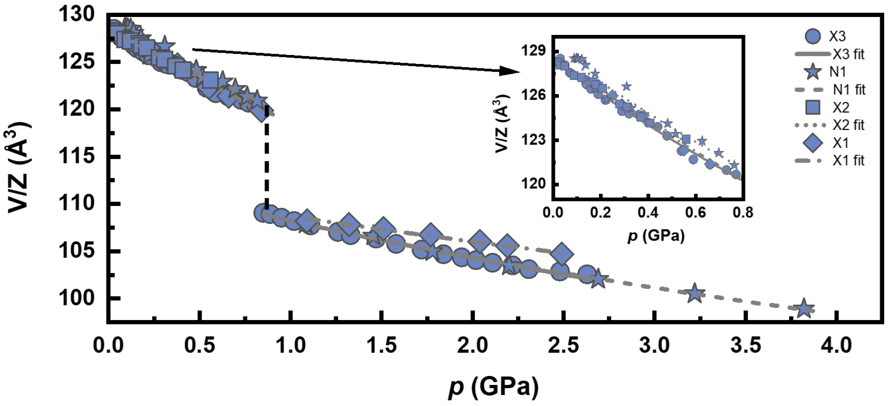

Figure S4: The volume of Cu(tcm) versus pressure at ambient temperature. The value for the tetragonal phase is normalised ($V = V \times \sqrt{2}$) (Eq S2) to make it easier to follow the change in crystal structure after the ferroelastic phase transition. Cu(tcm) shows a continuous unit cell volume per molecule (V/Z) between these two phases. In the plot, all data points in this figure were fitted to the second-order Birch-Murnaghan equation of state (EoS) using EoSFit7-GUI.[S3] The applicability of the second-order fit was determined by inspection of the f-F plots, which were used to visualise the normalised pressure vs. Eulerian strain data for each phase. A horizontal trend within the experimental error was observed in the f-F plots for all phases, indicating that a second-order Birch-Murnaghan fit was sufficient to account for the pressure-volume data.[S4] According to the fitting results of the pressure-volume data, the bulk modulus (B_{Pi}) and its pressure derivative (B') for each phase were calculated with a reasonable error, as shown in Table S1. Here, P_i represents the initial pressure at which each phase appears: ambient pressure for the tetragonal phase, 0.12 GPa for the orthorhombic phase, and 0.93 GPa for the monoclinic phase.

Table S1: Compressibility and bulk modulus of Cu(tcm) calculated within different pressure ranges.

		K_a (Tpa^{-1})	K_b (Tpa^{-1})	K_c (Tpa^{-1})	B_{Pi} (GPa)	B′	Range(GPa)	Data points
$I4_1md$	N1	0	2.38	14.40	38(21)	4	0.09 – 0.12	5
	X3	79.0	79.0	0.7	5.7(4)	4	0.03 – 0.08	4
	X2	44.74	44.74	-43.21	15(4)	4	0.01 – 0.05	4
Fdd2	N1	160(14)	-14.9(11)	-93(13)	10.8(6)	4	0.14 – 0.93	13
	X3	165(5)	-14(2)	-87(8)	10.6(3)	4	0.12 – 0.81	19
	X2	214(29)	-14(5)	-131(40)	11.3(5)	4	0.09 – 0.56	10
	X1	118(7)	-9.8(0)	-35(7)	10.2(5)	4	0.19 – 0.84	7
Cc	N1	-1.7(2)	27.1(8)	3.5(4)	22.3(7)	4	1.08 – 3.82	7
	X3	-1.1(1)	31.5(5)	1.1(3)	21.2(6)	4	0.93 – 2.63	16
	X1	0.14(10)	22.7(3)	3.5(3)	34(2)	4	1.08 – 2.49	7

* Due to the limited number of data points in the $I4_1md$ phase and its narrow stability range, accurate determination of directional linear compressibilities with reliable error margins was not possible for this phase. The values presented should be considered as approximate indicators

3. Crystallographic Details

Table S2: Crystallographic details determined by Rietveld refinement of N1 data for Cu(tcm) at 0.09 GPa.

Space group	*$I4_1md$*
a / Å	7.5433(2)
c / Å	9.0347(10)
β/ °	90
V / $Å^3$	514.08(6)
R_{wp}	6.33%

**Atom**	**occ**	**x**	**y**	**z**	**B_{iso}* / $Å^2$**
C1	1	0	0	0.462(7)	4.1(5)
C2	1	0	0	0.124(6)	4.1(5)
C3	1	0.370(7)	0	0.636(4)	4.1(5)
Cu1	1	0	0	0	4.1(5)
N1	1	0	0	0.236(6)	4.1(5)
N2	1	0.262(5)	0	0.571(6)	4.1(5)

* B_{iso} constrained to be equal for all atoms.

Table S3: Crystallographic details determined by Rietveld refinement of N1 data for Cu(tcm) at 0.18 GPa.

Space group	*Fdd2*
a / Å	10.5254(10)
b / Å	10.7067(11)
c / Å	9.0522(8)
β/ °	90
V / Å3	1020.12(17)
Rwp	4.06%

**Atom**	**occ**	**x**	**y**	**z**	**Biso* / Å2**
C1	1	0	0	0.470(6)	2.8(3)
C2	1	0	0	0.327(7)	2.8(3)
C3	1	0.665(5)	0.195(4)	0.335(6)	2.8(3)
Cu1	1	0	0	0	2.8(3)
N1	1	0	0	0.215(5)	2.8(3)
N2	1	0.617(4)	0.094(3)	0.389(5)	2.8(3)

* B_{iso} constrained to be equal for all atoms.

Table S4: Crystallographic details determined by Rietveld refinement of N1 data for Cu(tcm) at 1.78 GPa.

Space group	*Cc*
a / Å	9.695(2)
b / Å	9.4685(7)
c / Å	5.6459(10)
β/ °	125.86(2)
V / Å^3	420.07(16)
R_{wp}	3.55%

Atom	occ	x	y	z	B_{iso}* / Å^2
C1	1	0.508(6)	0.123(3)	0.115(7)	0.53(11)
C2	1	0.337(3)	0.099(6)	0.004(8)	0.53(11)
C3	1	0.151(3)	0.408(5)	0.832(8)	0.53(11)
C4	1	-0.023(5)	0.296(3)	0.342(6)	0.53(11)
Cu1	1	-0.012(5)	0.126(3)	-0.077(5)	0.53(11)
N1	1	0.212(3)	0.095(3)	-0.031(8)	0.53(11)
N2	1	0.785(4)	0.071(2)	0.558(6)	0.53(11)
N3	1	0.019(3)	0.240(2)	0.214(5)	0.53(11)

* B_{iso} constrained to be equal for all atoms.

4. X3 Data of Cu(tcm)

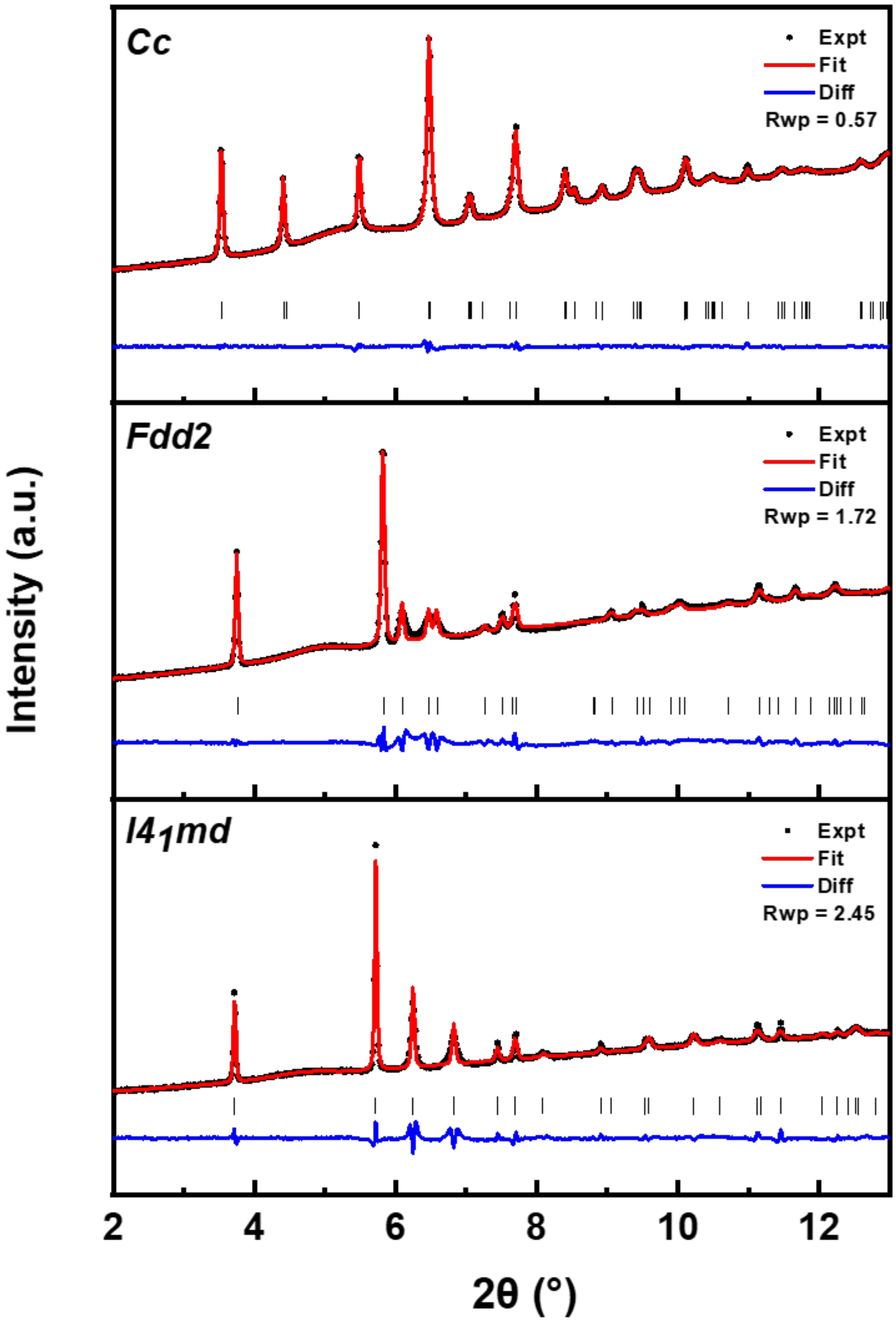

Figure S5: Powder X-ray diffraction patterns (X3) of the tetragonal (bottom), orthorhombic (middle), and monoclinic (top) phases of Cu(tcm) at 0.03 GPa, 0.30 GPa, and 1.26 GPa, respectively. They were fitted using Rietveld refinement. The experimental data are displayed in black, the fit in red, the residuals in grey, and the permitted reflections are denoted by vertical bars. The Rwp value is given as a satisfactory indicator of refinement. In the tetragonal ($I4_1md$) phase pattern, clear peak broadening indicates significant strain-induced anisotropy, leading to the transformation into an orthorhombic (*Fdd2*) structure.

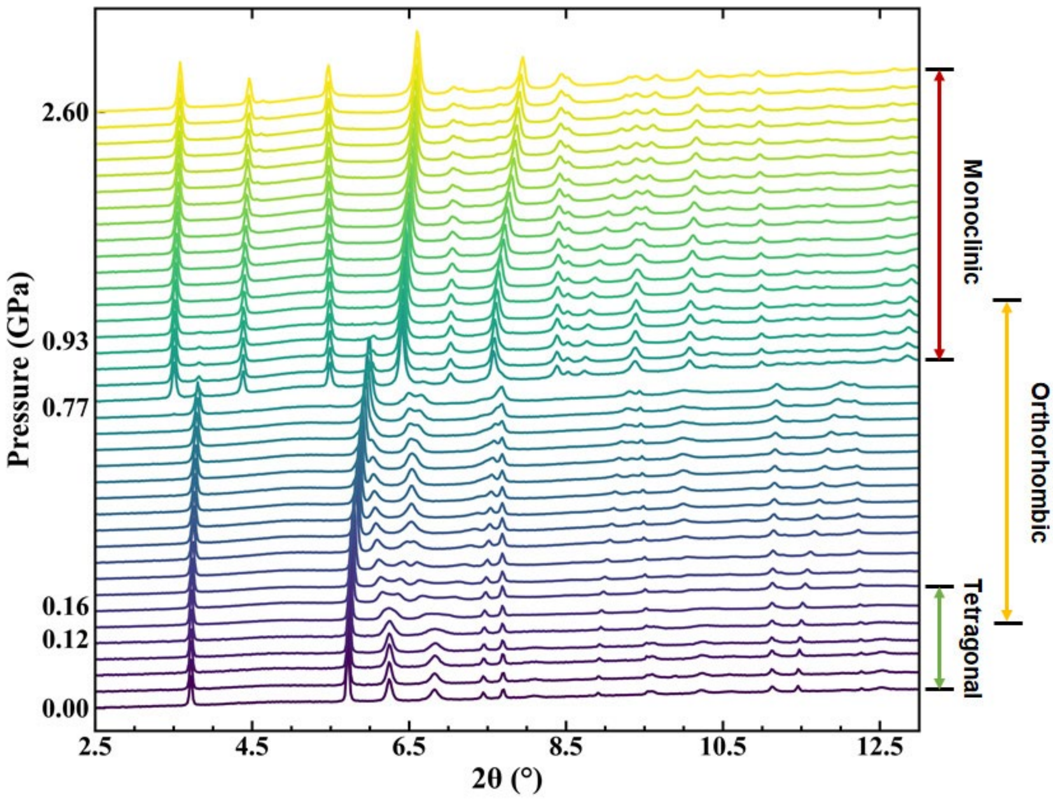

Figure S6: HP-PXRD patterns of Cu(tcm) measured at Diamond Light Source (X3) using radiation with a wavelength of 0.3757 Å. The patterns represent, in ascending order, the tetragonal (0-0.12 GPa), orthorhombic (0.12-0.93 GPa) and monoclinic (0.77-2.6 GPa) phases.

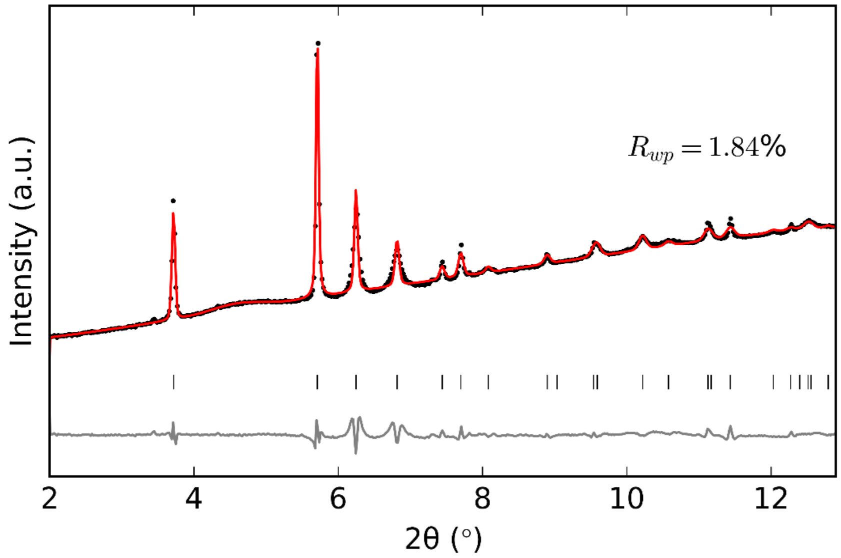

Figure S7: Powder X-ray diffraction pattern (X3) of the tetragonal phases of Cu(tcm) taken after decomposition. It was fitted using Rietveld refinement. The experimental data are shown in black, the fit in red, the residuals in grey, and the allowed reflections are indicated by vertical bars. The Rwp value is given as a satisfactory indicator of refinement.

Table S5: Lattice parameters and unit cell volumes (non-normalized) of Cu(tcm) from X3 dataset as a function of pressure during compression, except for the last point which was collected during decompression. Pressure uncertainties are estimated at ±0.1 GPa or less, with smaller uncertainties for fine pressure steps.

p / GPa	Space group	*a* / Å	*b* / Å	*c* / Å	*β* / °	*V* / Å^3
0.03	$I4_1md$	7.535(4)	7.535(4)	9.054(4)	90	514.0(6)
0.05	$I4_1md$	7.524(5)	7.524(5)	9.050(5)	90	512.3(7)
0.07	$I4_1md$	7.511(5)	7.511(5)	9.044(6)	90	510.3(7)
0.08	$I4_1md$	7.506(5)	7.506(5)	9.050(6)	90	509.9(8)
0.12	*Fdd2*	10.421(5)	10.787(5)	9.055(3)	90	1017.9(7)
0.14	*Fdd2*	10.339(4)	10.842(5)	9.055(3)	90	1015.1(7)
0.16	*Fdd2*	10.188(5)	10.964(6)	9.056(3)	90	1011.6(8)
0.19	*Fdd2*	10.080(5)	11.048(6)	9.059(3)	90	1008.9(8)
0.22	*Fdd2*	9.942(5)	11.159(6)	9.064(4)	90	1005.7(9)
0.29	*Fdd2*	9.792(4)	11.261(7)	9.066(4)	90	999.7(9)
0.32	*Fdd2*	9.720(4)	11.311(6)	9.080(4)	90	998.3(8)
0.36	*Fdd2*	9.677(2)	11.338(3)	9.093(2)	90	997.7(5)
0.40	*Fdd2*	9.530(3)	11.486(5)	9.076(3)	90	993.5(6)
0.44	*Fdd2*	9.490(2)	11.490(4)	9.090(3)	90	991.2(5)
0.48	*Fdd2*	9.435(2)	11.493(3)	9.096(2)	90	986.4(4)
0.54	*Fdd2*	9.355(4)	11.493(5)	9.097(3)	90	978.2(7)
0.59	*Fdd2*	9.294(2)	11.505(3)	9.106(3)	90	973.6(5)
0.66	*Fdd2*	9.230(2)	11.528(3)	9.125(3)	90	970.9(5)
0.73	*Fdd2*	9.166(5)	11.557(4)	9.137(4)	90	967.9(8)
0.77	*Fdd2*	9.083(4)	11.613(5)	9.153(5)	90	965.5(8)
0.81	*Fdd2*	9.051(6)	11.635(9)	9.154(8)	90	964.0(13)
0.85	*Cc*	9.653(4)	9.827(2)	5.6606(9)	125.67(2)	436.19(2)
0.89	*Cc*	9.652(3)	9.8094(14)	5.6599(7)	125.67(2)	435.67(2)
0.93	*Cc*	9.652(2)	9.7844(11)	5.6582(5)	125.657(12)	434.19(13)
1.02	*Cc*	9.651(2)	9.7537(11)	5.6574(4)	125.643(13)	432.80(14)
1.11	*Cc*	9.650(2)	9.7125(11)	5.6559(5)	125.625(12)	430.88(13)
1.26	*Cc*	9.650(2)	9.6520(11)	5.6542(5)	125.609(12)	428.18(14)
1.33	*Cc*	9.649(3)	9.6259(13)	5.6519(5)	125.603(11)	426.82(15)
1.47	*Cc*	9.6525(13)	9.5837(7)	5.6533(5)	125.572(10)	425.37(9)
1.58	*Cc*	9.650(2)	9.5423(8)	5.6493(6)	125.570(15)	423.12(12)
1.72	*Cc*	9.646(2)	9.4893(9)	5.6469(6)	125.520(13)	420.70(12)
1.84	*Cc*	9.644(2)	9.4454(9)	5.6450(7)	125.487(13)	418.68(11)
1.94	*Cc*	9.6413(15)	9.4175(9)	5.6439(7)	125.458(12)	417.41(11)
2.02	*Cc*	9.642(2)	9.3913(9)	5.6423(7)	125.449(13)	416.21(12)
2.11	*Cc*	9.6421(15)	9.3662(10)	5.6412(7)	125.429(13)	415.12(11)
2.22	*Cc*	9.644(2)	9.3382(10)	5.6407(8)	125.402(14)	414.06(12)
2.31	*Cc*	9.640(2)	9.3074(11)	5.6371(8)	125.38(2)	412.41(13)
2.48	*Cc*	9.641(2)	9.279(2)	5.6370(9)	125.327(14)	411.42(13)
2.63	*Cc*	9.641(2)	9.2463(11)	5.6366(8)	125.279(13)	410.18(13)
0.00	$I4_1md$	7.541(5)	7.541(5)	9.037(6)	90	513.9(7)

5. N1 Data of Cu(tcm)

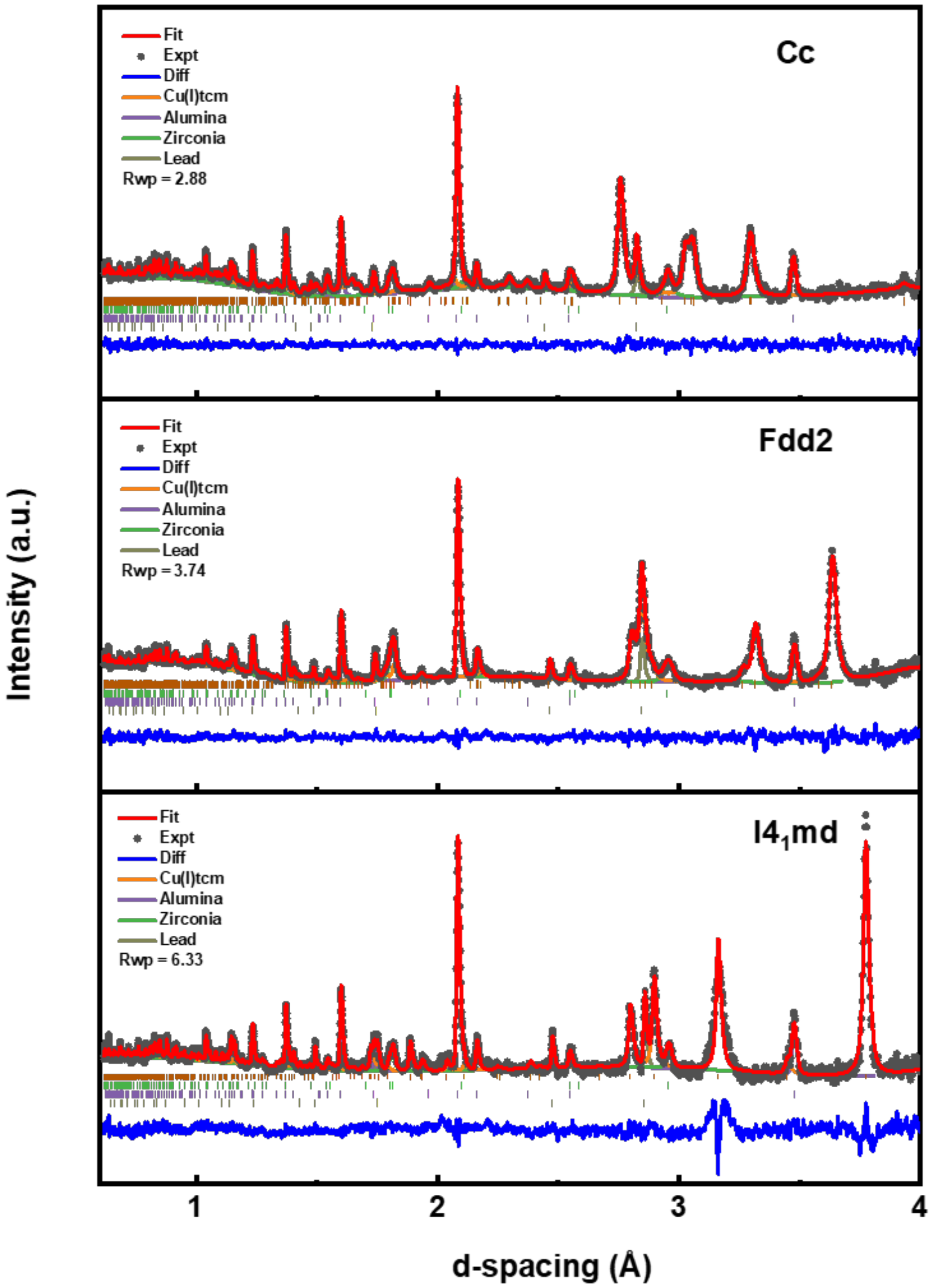

Figure S8: Time of flight (ToF) neutron diffraction pattern (N1) of the tetragonal (bottom), orthorhombic (middle), and monoclinic (top) phases of Cu(tcm) at 0.09 GPa, 0.63 GPa, and 1.78 GPa, fitted using Rietveld refinement. The experimental data are displayed in black, the fit in red, and the residuals in blue. The calculated signals for alumina, zirconia, and lead, which come from anvil and pressure marker, are represented by purple, green, and dark green lines, respectively, while the permitted reflections are represented by vertical bars. The R_{wp} value is given as a satisfactory indicator of refinement.

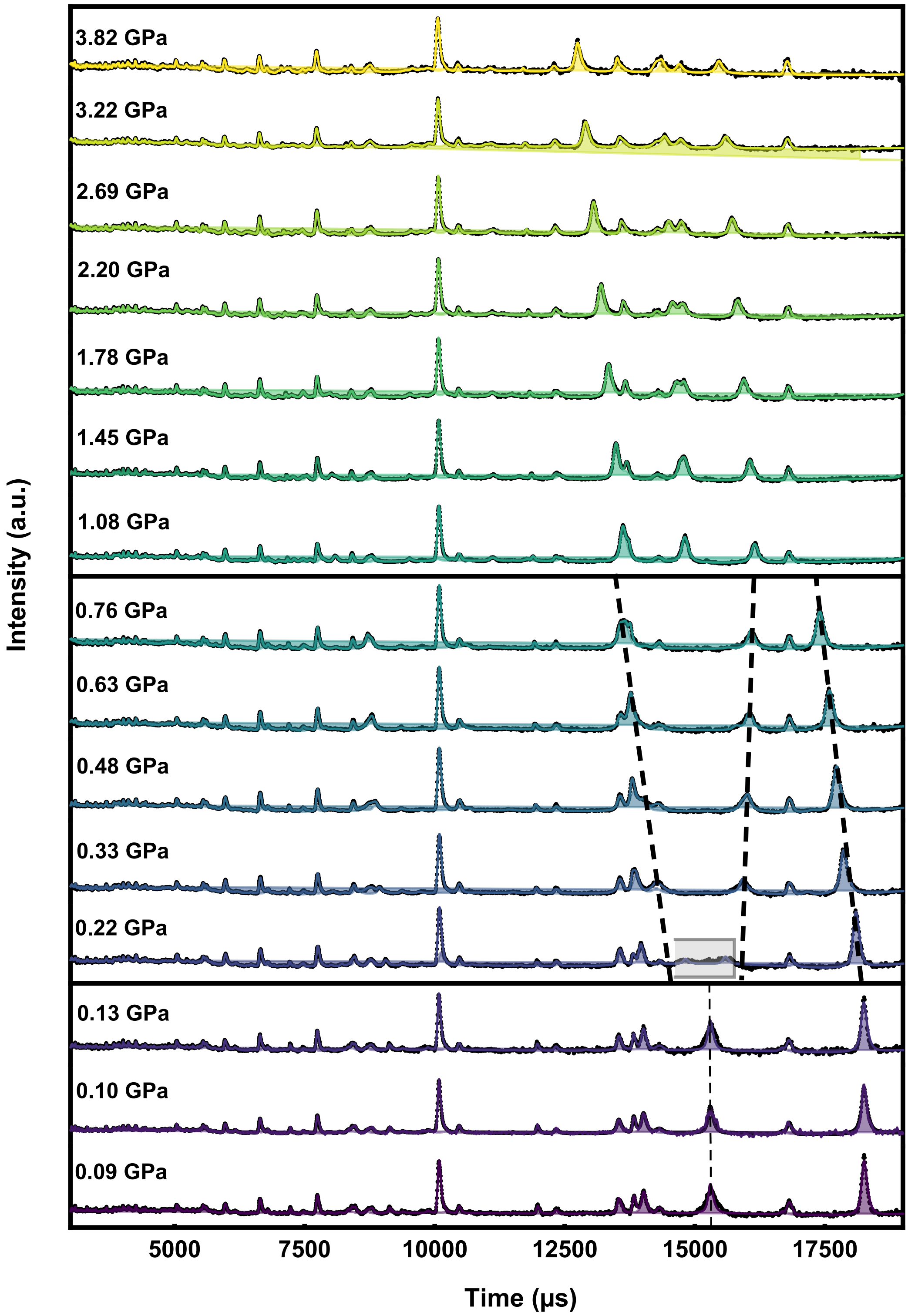

Figure S9: High-pressure neutron diffraction patterns (N1) of Cu(tcm), fitted using Rietveld refinement. The experimental data are represented by black dots, while the fittings are represented by sequential lines. The calculated signals for Cu(tcm) are displayed as patterns of the same hue as the fitting lines.

Table S6: Lattice parameters and unit cell volumes (non-normalized) of Cu(tcm) from N1 dataset as a function of pressure during compression.

p / GPa	Space group	*a* / Å	*b* / Å	*c* / Å	*β* / °	*V* / Å^3
0.089(9)	$I4_1md$	7.5433(2)	7.5433(2)	9.0347(10)	90	514.08(6)
0.103(10)	$I4_1md$	7.5428(3)	7.5428(3)	9.0330(12)	90	513.92(8)
0.132(11)	$I4_1md$	7.5403(3)	7.5403(3)	9.0336(12)	90	513.62(8)
0.137(12)	*Fdd2*	10.5848(11)	107007(11)	9.0448(11)	90	1024.5(2)
0.176(11)	*Fdd2*	10.5255(10)	10.7067(11)	9.0522(8)	90	1012.1(2)
0.178(2)	*Fdd2*	10.534(2)	10.690(2)	9.0579(14)	90	1020.0(3)
0.225(12)	*Fdd2*	10.223(2)	10.967(3)	9.0614(9)	90	1015.9(3)
0.309(11)	*Fdd2*	10.030(2)	11.140(2)	9.0664(11)	90	1013.0(3)
0.330(9)	*Fdd2*	9.7088(12)	11.358(2)	9.0808(10)	90	1001.4(2)
0.482(7)	*Fdd2*	9.5021(8)	11.4917(13)	9.0940(9)	90	993.0(2)
0.516(10)	*Fdd2*	9.4144(10)	11.5235(15)	9.1014(11)	90	987.4(2)
0.627(2)	*Fdd2*	9.3532(10)	11.540(2)	9.1118(14)	90	983.5(2)
0.697(11)	*Fdd2*	9.2406(10)	11.5883(14)	9.124(2)	90	977.1(2)
0.762(11)	*Fdd2*	9.1525(12)	11.619(2)	9.126(2)	90	970.5(3)
0.816(14)	*Fdd2*	9.0746(14)	11.647(2)	9.153(2)	90	967.4(3)
0.926(10)	*Fdd2*	9.0064(12)	11.667(2)	9.152(2)	90	961.7(3)
1.083(4)	*Cc*	9.682(4)	9.7206(8)	5.6520(11)	125.78(3)	431.55(3)
1.455(13)	*Cc*	9.684(3)	9.5993(6)	5.6536(8)	125.75(3)	426.5(2)
1.78(2)	*Cc*	9.658(3)	9.4694(7)	5.6444(11)	125.54(3)	419.9(2)
2.20(2)	*Cc*	9.662(4)	9.3399(7)	5.6339(10)	125.49(2)	413.9(2)
2.69(3)	*Cc*	9.661(3)	9.2127(7)	5.6253(10)	125.38(2)	408.2(2)
3.22(3)	*Cc*	9.653(3)	9.0757(9)	5.6165(14)	125.19(2)	402.1(2)
3.82(5)	*Cc*	9.648(4)	8.9510(12)	5.590(2)	124.96(3)	395.7(2)

6. X2 Data of Cu(tcm)

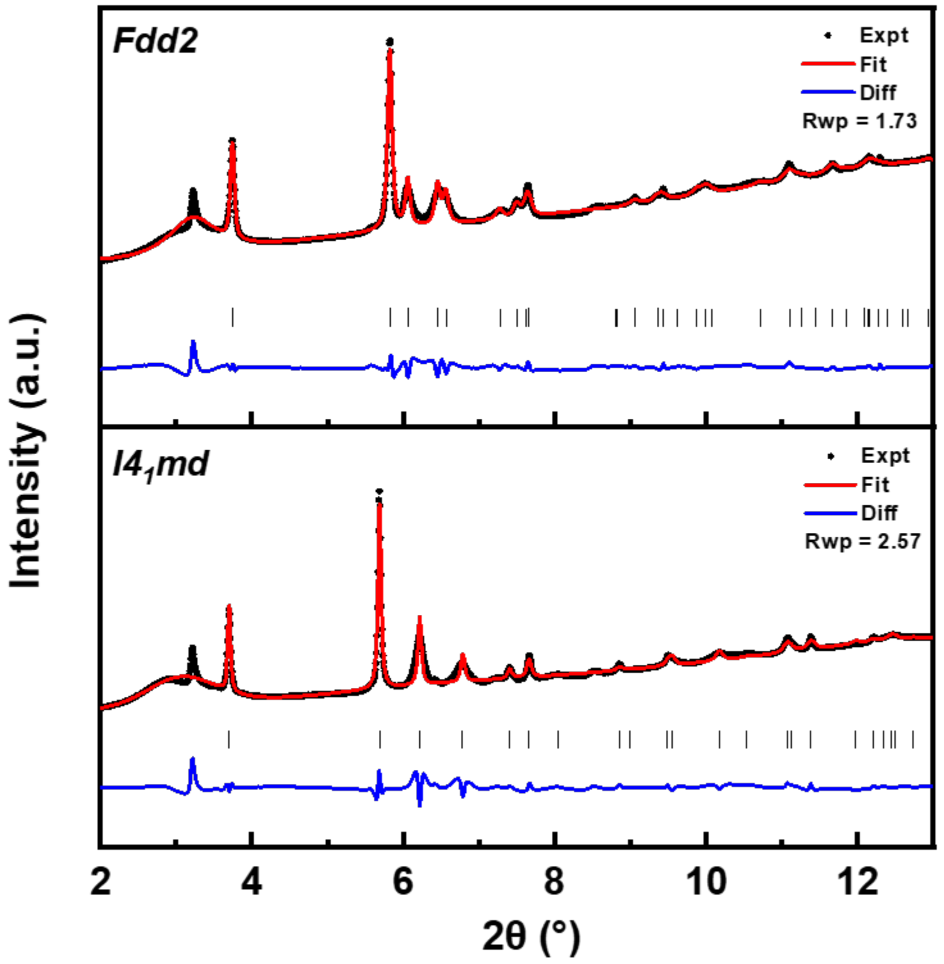

Figure S10: Powder X-ray diffraction patterns (X2) of the tetragonal (bottom) and orthorhombic (top) phases of Cu(tcm) at 0.01 GPa and 0.41 GPa, respectively. They were fitted using Rietveld refinement. The experimental data are displayed in black, the fit in red, the residuals in grey, and the permitted reflections are denoted by vertical bars. The Rwp value is given as a satisfactory indicator of refinement. The explanation of the extra peak can be found in SI Page 3.

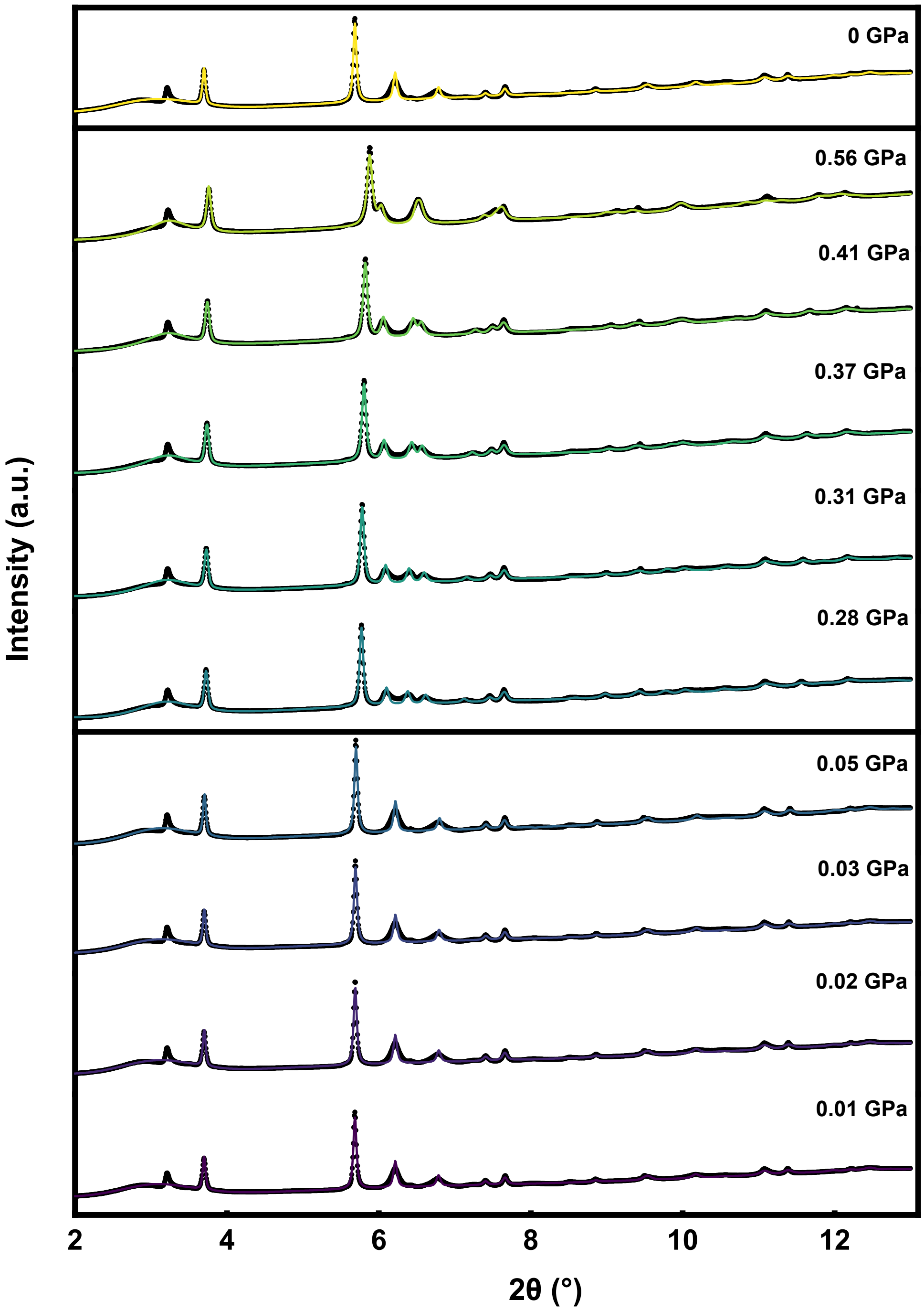

Figure S11: HP-PXRD patterns (X2, λ = 0.3738 Å) of Cu(tcm), fitted using Rietveld refinement. The patterns represent, in ascending order, the tetragonal phase, orthorhombic phase, and the recovered tetragonal phase after decompression.

Table S7: Lattice parameters and unit cell volumes (non-normalized) of Cu(tcm) from X2 dataset as a function of pressure during compression, except for the last point which was collected during decompression. Pressure uncertainties are estimated at ±0.1 GPa or less, with smaller uncertainties for fine pressure steps.

p / GPa	Space group	*a* / Å	*b* / Å	*c* / Å	*β* / °	*V* / Å^3
0.01	$I4_1md$	7.535(8)	7.535(8)	9.037(8)	90	513.1(11)
0.02	$I4_1md$	7.532(8)	7.532(8)	9.043(8)	90	513.0(11)
0.03	$I4_1md$	7.527(8)	7.527(8)	9.043(9)	90	512.3(12)
0.05	$I4_1md$	7.521(8)	7.521(8)	9.052(9)	90	512.0(13)
0.09	*Fdd2*	10.483(6)	10.742(6)	9.048(5)	90	1018.9(10)
0.12	*Fdd2*	10.457(6)	10.748(6)	9.058(5)	90	1017.9(10)
0.17	*Fdd2*	10.393(9)	10.775(9)	9.068(7)	90	1015.5(14)
0.18	*Fdd2*	10.354(9)	10.800(10)	9.070(8)	90	1014(2)
0.21	*Fdd2*	10.266(8)	10.864(9)	9.073(6)	90	1012.0(13)
0.28	*Fdd2*	9.984(7)	11.085(9)	9.068(6)	90	1003.6(13)
0.31	*Fdd2*	9.921(7)	11.134(8)	9.072(6)	90	1002.1(12)
0.37	*Fdd2*	9.791(7)	11.211(8)	9.078(6)	90	996.5(12)
0.41	*Fdd2*	9.722(7)	11.245(8)	9.086(6)	90	993.3(12)
0.56	*Fdd2*	9.537(8)	11.305(10)	9.133(7)	90	984.6(14)
0.10	$I4_1md$	7.534(8)	7.534(8)	9.039(9)	90	513.1(12)

7. X1 Data of Cu(tcm)

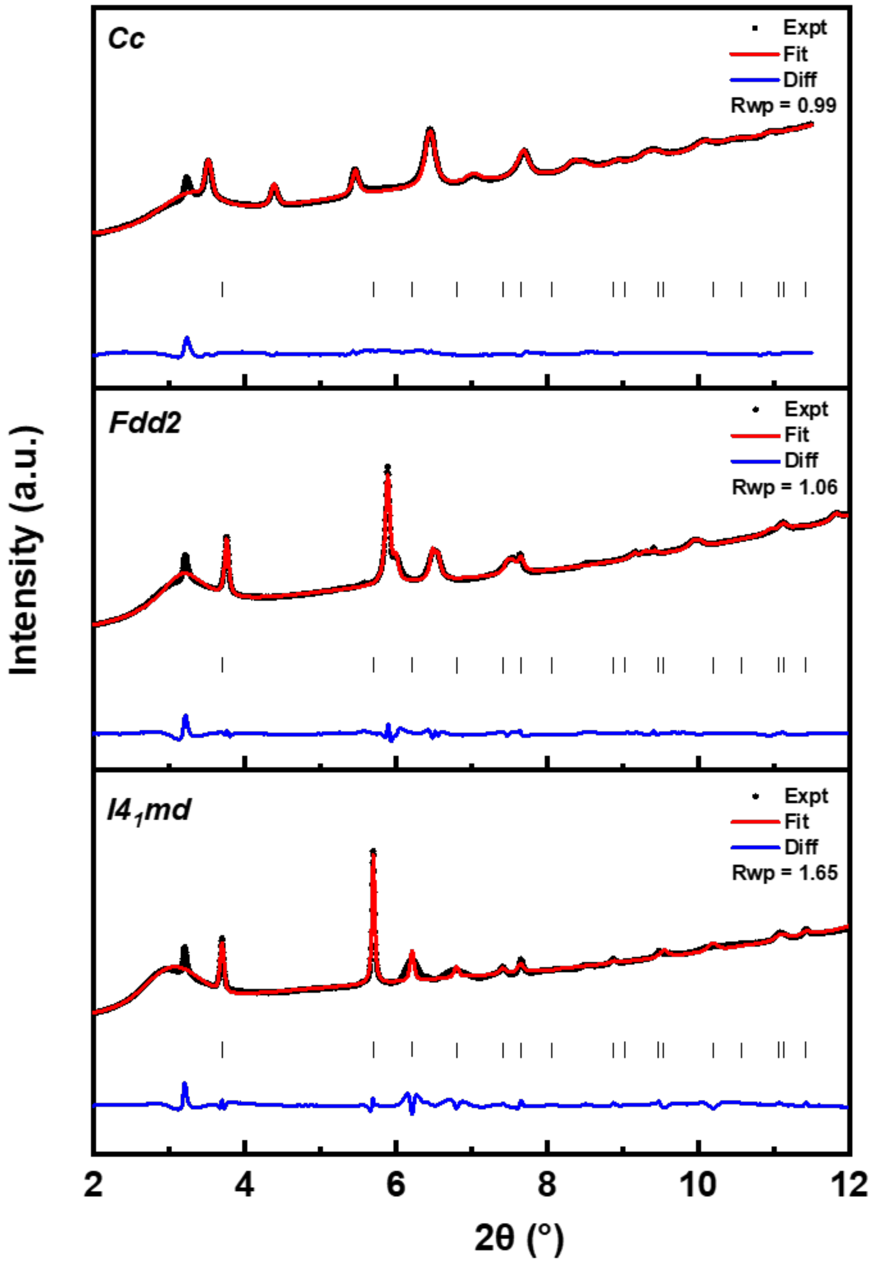

Figure S12: Powder X-ray diffraction patterns (X1) of the tetragonal (bottom), orthorhombic (middle), and monoclinic (top) phases of Cu(tcm) at 0.08 GPa, 0.55 GPa, and 1.77 GPa, respectively. They were fitted using Rietveld refinement. The experimental data are displayed in black, the fit in red, the residuals in grey, and the permitted reflections are denoted by vertical bars. The Rwp value is given as a satisfactory indicator of refinement. The explanation of the extra peak can be found in SI Page 3.

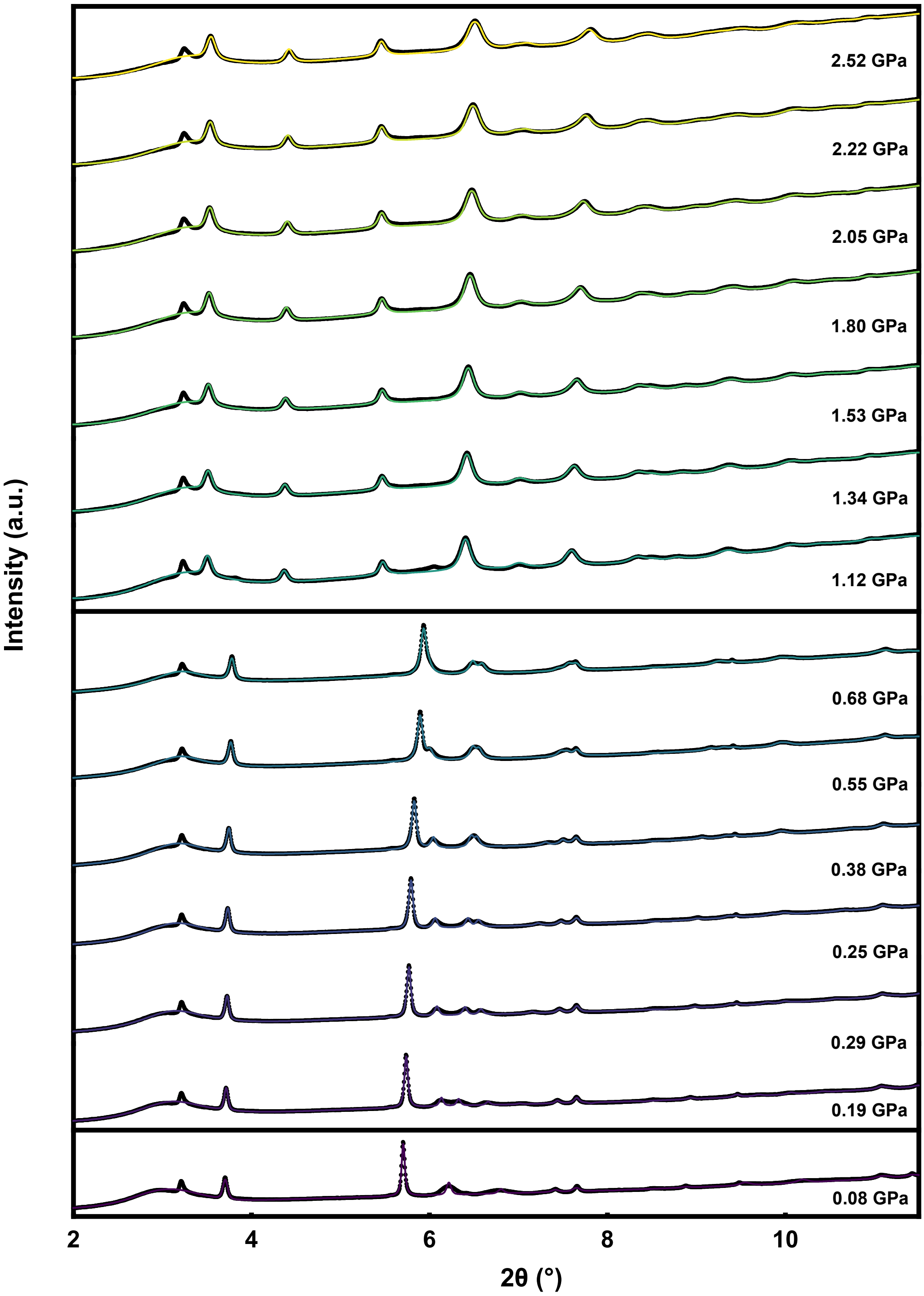

Figure S13: HP-PXRD patterns (X1, λ = 0.3738 Å) of Cu(tcm), fitted using Rietveld refinement. The patterns represent, in ascending order, the tetragonal, orthorhombic and monoclinic phases.

Table S8: Lattice parameters and unit cell volumes (non-normalized) of Cu(tcm) from X1 dataset as a function of pressure during compression. Pressure uncertainties are estimated at ±0.1 GPa or less, with smaller uncertainties for fine pressure steps.

p / GPa	Space group	*a* / Å	*b* / Å	*c* / Å	*β* / °	*V* / Å^3
0.08	$I4_1md$	7.511(10)	7.511(10)	9.059(11)	90	511(2)
0.19	*Fdd2*	10.196(4)	10.971(5)	9.063(3)	90	1013.8(7)
0.25	*Fdd2*	9.936(3)	11.186(4)	9.070(3)	90	1008.1(6)
0.29	*Fdd2*	9.814(3)	11.264(4)	9.076(3)	90	1003.3(5)
0.38	*Fdd2*	9.674(3)	11.330(3)	9.101(3)	90	997.5(5)
0.55	*Fdd2*	9.378(4)	11.476(5)	9.093(4)	90	978.6(7)
0.66	*Fdd2*	9.263(4)	11.501(4)	9.117(4)	90	971.2(7)
0.84	*Fdd2*	9.132(4)	11.506(6)	9.128(6)	90	959.0(9)
1.09	*Cc*	9.607(9)	9.754(7)	5.673(5)	125.48(6)	432.9(7)
1.32	*Cc*	9.611(8)	9.709(7)	5.673(5)	125.44(5)	431.3(7)
1.51	*Cc*	9.611(8)	9.669(6)	5.671(4)	125.39(5)	429.6(6)
1.77	*Cc*	9.603(7)	9.613(6)	5.666(4)	125.26(4)	427.0(6)
2.04	*Cc*	9.588(7)	9.553(6)	5.659(4)	125.11(4)	424.0(6)
2.19	*Cc*	9.580(7)	9.517(7)	5.656(5)	125.03(3)	422.3(6)
2.49	*Cc*	9.561(7)	9.453(7)	5.645(5)	124.83(3)	418.8(6)

8. Structural comparison between Ag(tcm) and Cu(tcm)

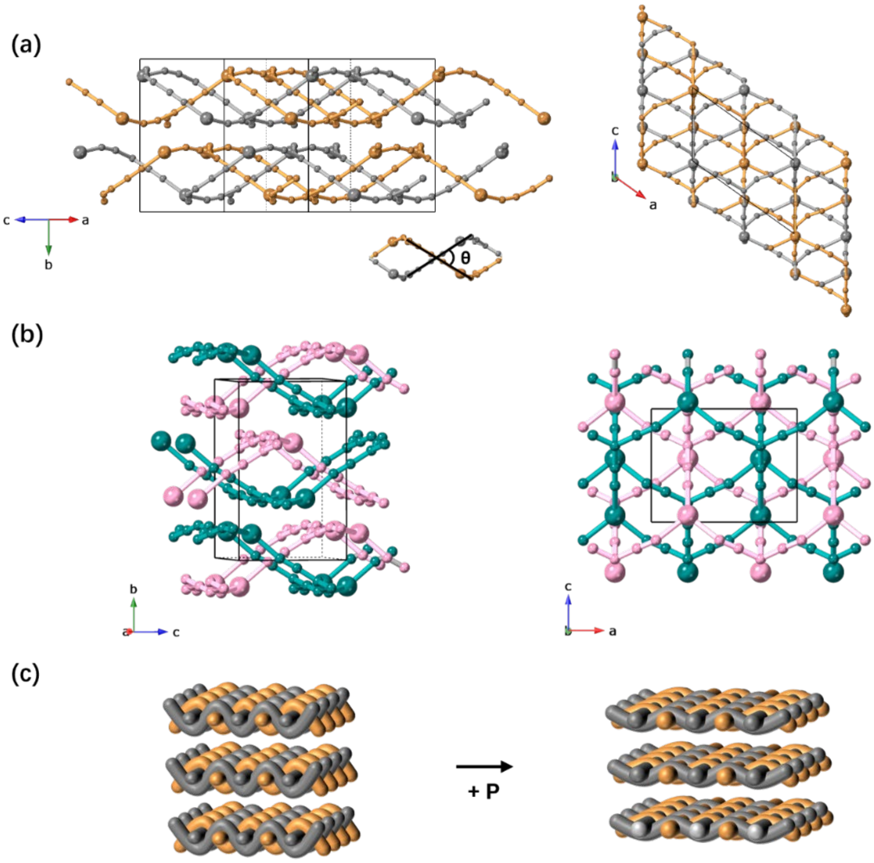

Figure S14: Structural comparison between monoclinic Cu(tcm) and Ag(tcm), highlighting structural similarities. (a) Crystal structure of Cu(tcm) viewed along two directions, with θ representing the inter-network angle. (b) Crystal structure of Ag(tcm) viewed along comparable directions. Both Cu(tcm) and Ag(tcm) show doubly-interpenetrating honeycomb networks. (c) Schematic mechanism illustrating how layer flattening under pressure leads to area expansion in the plane of the layers while decreasing interlayer distance.

The monoclinic phase of Cu(tcm) exhibits a layered structure with topologically double interpenetrating honeycomb networks, which shows remarkable similarities to the ambient structure of Ag(tcm). As shown in Figure S14, both materials feature stacked layers where metal ions (Cu^+ or Ag^+) and tcm^- ligands function as trigonal nodes, forming (6,3)-nets. These nets are doubly interpenetrated, creating a corrugated layer structure. The degree of layer corrugation is quantified by the inter-network angle θ.

The mechanism behind the negative area compressibility in Ag(tcm) can be understood through the pressure-driven dampening of layer rippling. As illustrated in panel (c), hydrostatic pressure causes the interlayer separation to decrease, but simultaneously flattens the corrugated layers by reducing θ. This flattening effect expands the cross-sectional area of each layer, counteracting the typical contraction expected under pressure. Unlike Ag(tcm), where the angle θ decreases from 62° to 61° upon compression, Cu(tcm) has an initial angle value of 61° after phase transition, and it remains constant with further compression. These phenomena explain the negative area compressibility observed in Ag(tcm) versus the zero area compressibility in Cu(tcm), and could be attributed to the higher structural symmetry in the former compared to the lower symmetry in the latter.

9. Reference

(S1) Cairns, A. B., Goodwin, A. L. "Negative linear compressibility." *Phys. Chem. Chem. Phys.* 2015, 17, 20449-20465.

(S2) Cliffe, M. J., Goodwin, A. L. "PASCal: a principal axis strain calculator for thermal expansion and compressibility determination." *J. Appl. Cryst.* 2012, 45, 1321-1329.

(S3) Angel, R. J., Alvaro, M., Gonzalez-Platas, J. "EosFit7c and a Fortran module (library) for equation of state calculations." *Z. Kristallogr. Cryst. Mater.* 2014, 229, 405-419.

(S4) Angel, R. J. "Equations of state." *Rev. Mineral. Geochem.* 2000, 41, 35-59.

(S5) Rietveld, H. M. "Line profiles of neutron powder-diffraction peaks for structure refinement." *Acta Cryst.* 1967, 22, 151-152.

(S6) Coelho, A. A. "TOPAS and TOPAS-Academic: an optimization program integrating computer algebra and crystallographic objects written in C++." *J. Appl. Cryst.* 2018, 51, 210-218.

(S7) Hunt, S. J., Cliffe, M. J., Hill, J. A., Cairns, A. B., Funnell, N. P., Goodwin, A. L. "Flexibility transition and guest-driven reconstruction in a ferroelastic metal–organic framework." *CrystEngComm.* 2015, 17, 361-369.

(S8) Hodgson, S. A., Adamson, J., Hunt, S. J., Cliffe, M. J., Cairns, A. B., Thompson, A. L., Tucker, M. G., Funnell, N. P., Goodwin, A. L. "Negative area compressibility in silver(I) tricyanomethanide." *Chem. Commun.* 2014, 50, 5264-5266.
